# An Examination of Subjective and Objective Measures of Stress in Tactical Populations: A Scoping Review

**DOI:** 10.3390/healthcare11182515

**Published:** 2023-09-11

**Authors:** Whitney Tramel, Ben Schram, Elisa Canetti, Robin Orr

**Affiliations:** 1 Faculty of Health Sciences and Medicine, Bond University, Robina, QLD 4226, Australia; bschram@bond.edu.au (B.S.); ecanetti@bond.edu.au (E.C.); rorr@bond.edu.au (R.O.); 2 Tactical Research Unit, Bond University, Robina, QLD 4226, Australia

**Keywords:** stress, occupational stress, tactical performance, military, law enforcement

## Abstract

Persons working in tactical occupations are often exposed to high-stress situations. If this stress is to be measured, an understanding of the stress outcomes used in these occupations is needed. The aim of this review was to capture and critically appraise research investigating subjective and objective outcome measures of physiological stress in tactical occupations. Several literature databases (PubMed, EMBASE, EBsco) were searched using key search words and terms. Studies meeting inclusion criteria were critically evaluated and scored by two authors using the Joanne Briggs Institute (JBI) critical appraisal tool. Of 17,171 articles, 42 studies were retained. The Cohen’s Kappa agreement between authors was 0.829 with a mean JBI Score of included studies of 8.1/9 ± 0.37 points. Multiple subjective and objective measures were assessed during a variety of high-stress tasks and environments across different occupations, including police officers, emergency service personnel, firefighters, and soldiers in the military. Common objective outcomes measures were heart rate, cortisol, and body temperature, and subjective measures were ratings of perceived exertion, and the Self Trait Anxiety Inventory. Often used in combination (i.e., subjective and objective), these outcome measures can be used to monitor stressors faced by tactical personnel undergoing on-the-job training.

## 1. Introduction

Tactical personnel are individuals who serve in law enforcement, military, firefighting and rescue professions and are exposed to training and work environments that are mentally and physically demanding [1]. Demands often faced by tactical personnel include exposure to extreme environments, maneuvering across difficult and unpredictable terrain, heavy load carriage, disaster response, evading human threats, evacuating casualties, and many other tasks that require intense physical labor in austere environments [1,2,3]. As such, the rigorous nature of the profession exposes individuals to multiple stressors that can have a negative impact on performance characteristics that are critical to the duties they must carry out [4,5,6].

Hans Selye was considered the first person to define “stress” and proposed that stress is present in any individual throughout a period of exposure to a nonspecific demand [7]. According to Selye’s General Adaptation Syndrome theory, when an individual is first exposed to a stressor, whether it be internal or external, the individual will experience a disruption in their physiological system. This disruption causes a shift in homeostasis and a disruption from normal physiological processes, in turn initiating a cascade of neural and hormonal responses, including an increase in activation of the sympathetic nervous system (SNS) and of the hypothalamic–pituitary–adrenal (HPA) axis [7,8]. The resulting response from the stressor leads to an increase in an individual’s metabolic, muscular, and cardiovascular response [9,10], a decrease in cortical arousal and cognitive processing, and a diminished ability to perceive exertion [11,12]. Following an acute response, the individual’s body will quickly return to homeostasis or a ‘resting state’. In high-stress environments, where the demands of the stressor often exceed normal physiological ranges, the regulatory mechanisms for survival begin to decrease and the body’s ability to regulate itself and return to homeostasis diminishes [7,8].

Tactical personnel are exposed to a number of high-stress situations and environments throughout the course of their career, ranging from daily operational demands to environmental extremes and intense physical stimuli. As a result of exposure to these stressors, critical biological and cognitive functions, important for tactical performance, are often significantly reduced [5,6]. Even the smallest performance decrement could potentially be life threatening to the individual and others around them. Therefore, a high level of performance while overcoming various threats is critical for successful achievement of task and mission objectives [1,3,4]. Additionally, the long-term health and resilience of these personnel are dependent upon their ability to tolerate these demands and respond to stressors over time. Long-term impacts of constant exposure to occupational stress can lead to a myriad of health problems, including stress-related diseases, such as cardiovascular disease [13,14]. These acute (short term) and chronic (long term) concerns demonstrate a need for stress identification and potential monitoring for tactical personnel if they are to perform optimally and maintain long-term health and wellbeing.

In order to have a greater understanding of the stressors personnel are exposed to, concurrent assessment and monitoring of multiple subjective and objective measures may be critical. Taylor et al. [6] emphasizes the need to administer studies in realistic environments and scenarios to help practitioners better understand the consequences of real-life exposures to high-stress situations. Employment of these types of studies, along with monitoring of operationally relevant measures, may help advance the development of treatment for stress-related diseases and disorders specific to this population in the long-term [15]. In the short-term, stress monitoring may help identify acute decreases in key performance indicators allowing individuals to better prepare for the environments they may find themselves in [16].

However, in order to identify and monitor stress, the outcome measures used in this regard, and within this population specifically, need to be profiled. Therefore, the aim of this review was to capture and critically appraise research investigating subjective and objective measures of physiological stress in tactical populations. The key objective of this review is to understand the optimal subjective and objective measures of stress within tactical populations.

## 2. Materials and Methods

### 2.1. Protocol and Registration

The protocol for this study was registered under the Open Science Framework (osf.io/kq83c) [17]. This scoping review was conducted following the PRISMA guidelines for Scoping Reviews (PRISMA ScR) [18].

### 2.2. Eligibility Criteria

Relevant keywords were guided by the research question framing the review and based off keywords of previously known publications in the field (see Table 1). After a pilot search was conducted within the PubMed database, search terms were affirmed before entry into a Polyglot Search Translator [19], which translated the PubMed search terms to those aligned with the EMBASE and Elton B. Stephens Company (EBSCO: inclusive of The Cumulative Index to Nursing and Allied Health Literature (CINAHL) and SPORTDiscus.) databases. Filters were applied in each database, where available (Table 1).

### 2.3. Information Sources

Only studies conducted within the last 15 years were included to ensure studies were pertinent to current stressors faced by tactical personnel. EndNote Software (v. 20.4.1, Clarivate Analytics, Philadelphia, PA, USA) was used to collate all identified studies and employed to detect and remove duplicate articles. Duplicate articles that were not automatically captured by the software were removed manually on identification.

### 2.4. Search

The search strings used in each database are outlined in Table 1, including filters that were used and any exclusion terms, where relevant.

### 2.5. Selection of Sources of Evidence

Once duplicates were removed, all remaining studies were reviewed by screening titles and abstracts to remove studies clearly not relevant to this review. The remaining articles were screened against inclusion and exclusion criteria. Inclusion criteria were: (A) research participants were tactical personnel, (B) study included a measure of objective or subjective stress, (C) study was in relation to an occupational task or operational duties, (D) subjects were adults 18 years or older, (E) study followed a quantitative design, (F) study was published after 2005, and (G) study was published in, or translatable to, the English language. All articles meeting inclusion criteria were then assessed and compared against exclusion criteria; these being (A) stress measures examined during cognitive assessments only or (B) fitness measures (i.e., leg strength, run times, etc.), used as predictors of stress instead of psychological or physiological markers or (C) conducted in extreme temperatures (<5 °C or >40 °C) or atypical environments (high altitude, microgravity).

Data Charting Processes The characteristics of the studies including stressor, duration of event, outcome measures, timepoints, and pre- and post-results are provided in Table 2 with detailed results provided in Supplemental Table S1. The data extracted from each article included author, title, nationality, participant demographics, outcome measures and their results, and JBI score.

### 2.6. Data Items

The data items of interest include author, year, country, demographics of participants, identification of stressful event, duration of event, outcome measures, timepoints, pre- and post-values, and significance in *p* values. (Table 2 and Table 3).

### 2.7. Critical Appraisal of Individual Sources of Evidence

Following the search and screening process, eligible publications were assessed for methodological quality using the relevant Joanna Briggs Institute (JBI) Critical Appraisal Tool [58] dependent on study design. Two authors (WT and RO) assessed methodological quality independently and the agreement between them was assessed using Cohen’s Kappa by another reviewer (EC). A fourth reviewer (BS) was used to settle any disagreements on first-round scoring by acting as the third decisive score. The JBI critical appraisal tool includes a series of questions addressing the internal validity of a study and helps to determine the extent to which an article has addressed the possibility of bias in its design, conduct and analysis [59].

The JBI checklist for quasi-experimental studies was used. The checklist contained a total of eight questions depending on study type. The questions assessed the study’s sample, validity, and reliability of measurement of the exposure and outcomes, the criteria used for measurement of the condition, identification of confounding factors, and appropriate use of statistical analysis. Questions were scored on a binary scale of ‘1’ for those answered as ‘yes’, or ‘0’ for those answered as ‘no’, ‘unclear’, or ‘not applicable.’ The scores were added and divided by the number of questions in each checklist to provide a percentage and serve as a critical appraisal score.

### 2.8. Synthesis of Results

Stressors were profiled and results were grouped by subjective or objective measures and discussed based on emerging themes that were found (increasing, decreasing or no change).

## 3. Results

### 3.1. Selection of Sources of Evidence

**Figure 1:**
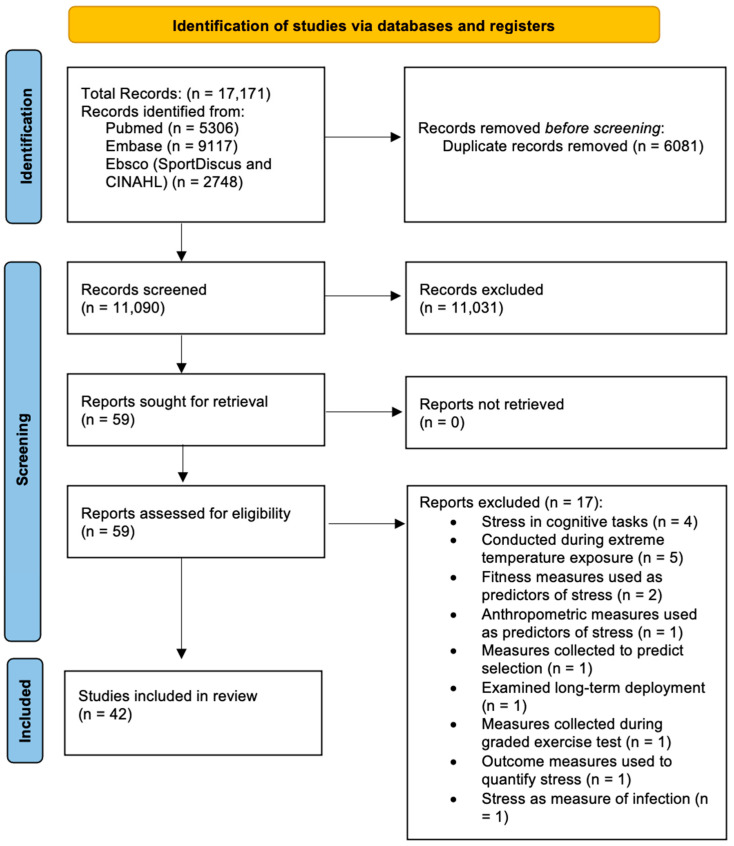
PRISMA flow diagram [61] detailing the systematic identification and screening of papers.

The results of the search, screening and selection process are detailed in the Preferred Reporting Items for Systematic Reviews and Meta-Analyses (PRISMA) [18] flow diagram (Figure 1). A total of 17,171 publications were captured in the initial search, following which 6020 duplicates were removed automatically with an additional 61 duplicates removed manually. Review by title and abstract removed 10,919 articles that clearly did not meet the review topic (e.g., relating to immunization in Navy personnel [60]) with another 112 articles excluded for not meeting the inclusion criteria. Of the 59 studies meeting the inclusion criteria, seventeen were removed for meeting the exclusion criteria. Following this process, 42 studies remained to inform this review.

### 3.2. Critical Appraisal

The mean CASP score was 8.1/9 ± 0.37 (range 7–9). The level of agreement between the two authors, as measured by Cohen’s Kappa (k = 0.829), was considered as an ‘almost perfect’ agreement [61].

#### 3.2.1. Study Design

Spain and the USA accounted for the largest number of studies, each having 12 included in this review. Five studies were from Finland [33,40,41,51,52], three from Japan [31,32,48], two from Australia [23,30] and two from Canada [44,53]. One study each came from Germany [20], Norway [27], France [36], Italy [42], Brazil [55] (also affiliated with the USA), the United Kingdom [56], and Iran [57].

#### 3.2.2. Demographics

Thirty-six studies included male participants only. The remaining six articles included both male and female participants [20,22,23,29,34,36]. Three tactical subpopulations were represented: the largest being military populations, with 26 articles. Twelve studies followed fire or rescue personnel [20,29,30,33,34,36,42,43,48,55,56,57]. Three studies followed police officers [22,32,37], while one investigated police recruits [44].

#### 3.2.3. Stressor Profile

The studies included in this review investigated a variety of stressful environments in different professions, including police officers, emergency service personnel, firefighters, and soldiers in the military. Shiftwork was examined in police [22,32], emergency personnel [20], and firefighters [33,48]. Outside of the regular stressors that these professions are exposed to, shiftwork provides additional strain on an individual, as it increases the time for exposure to stressors as well as negatively affecting the sleep cycle—leading to adverse health consequences from dysregulation of the HPA axis [22,32]. In police officers, researchers also examined the stress response to high fidelity simulation of policing events [44] and the effects of personal protective equipment during an occupational physical ability test (OPAT) [37]. Additionally, in firefighters, multiple live-fire training scenarios were investigated both with and without personal protective equipment (PPE) and self-contained breathing apparatus (SCBA) masks [29,30,34,36,42,43,55,56,57]. Military personnel were examined during combat simulations [24,26,46,47,49,50,62,63], cargo flights and parachute jumps [21,25,31,45], underwater evacuation training [54], selection courses [27,28,35,39], and longer term (5+ days in length) military field training [15,16,23,38,40,41,51,52,53,64]. Every operational task and training environment reported resulted in an increased level of stress, observed through both objective and subjective measures.

### 3.3. Objective Stress Measures

#### 3.3.1. Heart Rate and Measures of HRV

Twenty-four studies examined changes in heart rate (bpm) throughout the operational task or duty being performed [15,24,26,28,29,30,33,34,36,37,43,44,45,46,47,48,49,50,54,55,56,57,62,63], with 19 reporting significant findings [20,22,24,26,29,31,34,36,37,42,43,46,47,49,55,57,60,62,63]. In all studies with significant findings, mean HR increased from pre- to post-task (from 73.5 bpm to 110.8 bpm) (*p* ≤ 0.000). In the remaining five studies that did not report significant findings, a consequent increase in HR was still observed. One study specifically examined heart rate recovery (HRR) [36] by taking the difference between the exercise final HR and HR at 60 s and 300 s following three rescue interventions when compared to an incremental fitness test in firefighters. Researchers found that HRRs at 60 s and 300 s were significantly lower (*p* < 0.01) in firefighting tasks and interventions when compared to the incremental fitness test, showing that greater parasympathetic reactivation was observed following firefighting tasks.

Eleven studies utilized heart rate variability (HRV) as an assessment of physiological stress [26,33,36,37,45,47,48,49,54,62,63]. Only one study did not produce significant results [26]. Measures of HRV that were assessed include RMSSD, HF, LF, and R-R intervals. Decreases in RMSSD and HF values are a result of the increased sympathetic activity observed under stress. An overall significant decrease in RMSSD was observed following an operational task or maneuver [33,36,45,47,49] with the exception of findings from Tornero-Aguilera and Vicente-Rodriquez [54,63], where significant increases in RMSSD were observed. Significant HF values were observed in five studies, with three studies finding significant decreases [37,45,47] following combat maneuvers, and two finding significant increases [54,63]. Additionally, four studies found significant increases in LF values [45,47,49,63], while two found a significant decrease from baseline to post-maneuver [37,54]. Clemente-Suarez et al. [62] assessed average R-R intervals and observed significant decreases following combat simulations.

#### 3.3.2. Blood Lactate

Blood lactate concentration ([La^−^]_b_) was examined in 11 studies [21,24,37,45,46,47,49,50,53,62,63]. Ten studies identified a significant increase in [La^−^]_b_ during occupational tasks or duties, ranging from an increase of 3.6 mmol/L [21] to an increase of 12.08 mmol/L [49]. One article found significant rises in blood lactate following night and instrument helicopter flights [21], while another observed significant differences in blood lactate between loaded and unloaded maneuvers, with the unloaded condition resulting in greater values [37]. Two articles found significant rises in blood lactate concentration following parachute jumps [24,45]; two articles examined differences in blood lactate in high versus low trained groups, with both groups resulting in significant increases following high-stress simulations [50,63]; and four articles observed significant rises in blood lactate following melee combat simulations [46,47,49,62].

#### 3.3.3. Blood Oxygen Saturation

Of the 10 studies that investigated blood oxygen saturation (SpO_2_) as a measure of physiological stress [21,24,28,45,46,47,49,50,54,63], six (REFS) found a significant change in SpO_2_ following a stressful event. Only one study observed a significant increase in SpO_2_ following a special operations selection course [28], while another by Tornero-Aguilera et al. observed a significant decrease in SpO_2_ following an underground combat operation [49]. Significant decreases in SpO_2_ were also observed in two studies following a combat simulation both with and without a parachute jump [45,46]. Additionally, two studies examined differences in SpO_2_ following a combat simulation between higher- and lower-trained groups, with the lower-trained groups presenting with significant decreases is SpO_2_ when compared to those with more training [50,63].

#### 3.3.4. Creatine Kinase

Four studies examined levels of creatine kinase (CK) during occupational tasks [23,24,27,40]. Acute increases in CK are typically observed following strenuous exercise or training, while elevated levels could be a marker of muscle damage [65]. A significant increase in CK levels were observed in soldiers following prolonged military field training [40,41] and during ‘hell week’ in a Special Forces selection course [27]. The military field training was 22 days in length, while hell week was 7 days in length. In a study by Clemente-Suarez et al. [24], an increase in CK levels were observed in both novel and experienced warfighters following a parachute jump, but the results were not significant.

#### 3.3.5. Serum or Plasma Hormones

Twelve studies examined blood samples [15,23,27,35,38,39,40,41,51,52,53,64]. Eighteen different hormones from samples were examined as indicators of stress markers in tactical operators. These include cortisol, testosterone, IGF-1, SHBG, DHEA, IL-6, NPY, STfR, hepcidin, TNF-alpha, BDNF, epinephrine, norepinephrine, dopamine, C-reactive protein, TSH, leptin, T3 and T4. Significant increases were observed in serum cortisol [15,27,39,41,52,53,64], SHBG [27,41,51,52], epinephrine [15,64], norepinephrine [15,64], dopamine [64], DHEA [16,53], sTfR [15], IL-6 [38], C-reactive protein [27] and hepcidin [38] during operational tasks, all indicating in increase in stress. Furthermore, significant decreases were observed in serum testosterone [15,27,39,41,52,53,64], IGF-1 [39,40,41,52], TNF-alpha [40], leptin [40], T3 and T4 [27]. No significant findings were observed in serum BDNF and TSH.

#### 3.3.6. Salivary Hormones

Nine studies examined salivary samples [6,15,20,22,24,31,32,42,44]. Six different hormones from salivary samples were examined as indicators of stress markers in tactical operators. These include cortisol, alpha-amylase, C-reactive protein, NPY, DHEA, and testosterone. Significant increases were observed in salivary cortisol [15,16,20,22,32,42,44], DHEA [15,16], alpha-amylase [31,42], and NPY [15]. Significant decreases were observed in one study examining salivary testosterone [15]. No significant findings were observed in salivary C-reactive protein.

#### 3.3.7. Body Temperature

Thirteen studies examined body temperature as a measure of thermal stress during tactical operations [21,26,28,29,30,34,43,45,46,48,49,55,57]. Temperature was measured in three different ways: skin temperature via infrared thermometer [26,28,30,45,46,49,50,57], core temperature via disposable sensor capsules [29,30,34,43,55], and oral temperature with the use of a clinical thermometer [48]. Significant increases in body temperature during tasks were observed in six studies [29,30,34,43,55,57]. All studies that observed significant increases in body temperature were in firefighters during live fire tasks. The remaining seven studies observed body temperature during simulated military operations and tasks and found either no change or a small, nonsignificant decrease in temperature from baseline to immediately following completion of the task [21,26,28,45,46,48,49].

#### 3.3.8. Critical Flicker Fusion Threshold

Twelve studies examined cortical arousal through the Critical Flicker Fusion Threshold [21,24,28,45,46,47,48,49,50,54,62,63]. Only two studies observed significant decreases in cortical activation in soldiers following a simulated underground operation [49] and a special operations course [28], suggesting CNS fatigue and reduction in efficiency to process information. The remaining studies observed a decrease in cortical activation via use of the CFFT, although not approaching significance. Tasks included night and instrumental helicopter flights in pilots [21], parachute jumps [24,45], and combat simulations [46,47,50,54,62,63] in soldiers, and shift schedules in firefighters [48].

#### 3.3.9. Other

Three studies included objective outcome measures of stress that were only mentioned once. Hunt et al. [30] utilized a Physiological Strain Index (PSI), calculated based on time-aligned heart rate and core temperature measurements, and Adapted Physiological Strain Index (aPSI), which incorporated skin temperature into the calculation of the strain index. Both indices revealed increased levels of strain following firefighting training activities, with aPSI revealing significantly higher peak strain ratings.

The hemostatic response to an acute bout of fire training activities was assessed by Horn et al. [29], where significant increases in platelet count and in platelet closure time pre- to post-firefighting were observed. Lastly, Hormeno-Holgado [28] assessed urine color for dehydration and utilized the urine Combur-test to measure urine nitrates, protein, glucose, and pH in soldiers during a special operation selection course. Urine color, pH, and glucose all had significant negative changes from pre- to post-selection course, indicating individuals had undergone significant stress.

### 3.4. Subjective Stress Measures

#### 3.4.1. Ratings of Perceived Exertion (RPE)

Fourteen studies utilized RPE as a measure of perceived stress [21,26,28,36,37,40,43,45,46,49,54,56,62,63] with higher numbers corresponding to higher levels of perceived stress. Five studies reported a significant increase in RPE during and after combat simulation tasks [45,46,49,54,63]. Two studies specifically examined RPE during fire simulation tasks with and without breathing apparatus and found that RPE was significantly higher in tasks where SCBA masks are worn [26,36]. One study reported significantly greater ratings of perceived exertion during live firefighting tasks compared to free search tasks [56]. Bustamante-Sanchez et al. observed a significant increase in RPE in all instrument and night flights in Army aircrew members [21].

#### 3.4.2. State–Trait Anxiety Inventory (STAI)

Eleven studies used the State–Trait Anxiety Inventory as an assessment of state anxiety and trait anxiety [21,28,31,42,43,44,45,46,47,49,63]. Four studies identified significance from the questionnaire in relation to tasks operators were carrying out. Iizuka et al. [31] observed significantly higher state anxiety in pilots pre-flight compared to non-flight and post-flight. Two studies reported significantly higher increases in state anxiety in highly trained groups compared to lower-trained or lower-performing groups [44,63]. One study reported a significant decrease in state anxiety following a special operations course [28].

#### 3.4.3. Competitive State Anxiety Inventory (CSAI-2R)

Seven studies used the Competitive State Anxiety Inventory [21,24,45,46,47,49,63], which includes a self-evaluation of cognitive anxiety (CA), somatic anxiety (SA), and self-confidence (SC) on a scale of one (none at all) to four (very much). CA was significantly lower in experienced than novel warfighters in samples taken before and after a parachute jump [24]. Following a combat simulation, two studies observed significant decreases in CA [45,63], while one observed a significant increase [49]. Regardless of the task or experience, three studies observed significant decreases in SA [24,47,63], while three observed significant increases [45,46,49]. Self-confidence was significantly higher following a combat simulation with a parachute jump in experienced warfighters than novel [24].

#### 3.4.4. Profile of Mood States (POMS)

The Profile of Mood States was examined in four studies [15,23,42,53]. The POMS is an assessment showing changes in affective mood states (tension–anxiety, depression–dejection, anger–hostility, vigor–activity, fatigue–inertia, and confusion–bewilderment) on a five-point Likert scale. Chester et al. [23] used the POMS to assess baseline mental health status and residual psychological distress before and after environmental survival training (EST) and discovered significant decreases in vigor and fatigue from baseline through each phase of EST. Lieberman et al. [15] assessed POMS during simulated captivity in military survival training and observed significant increases in fatigue, confusion, tension, depression, anxiety, and total mood disturbance, while vigor significantly decreased from baseline throughout captivity, then recovering at the end, although not to baseline levels. Vartanian et al. [53] assessed the effect of military survival training on instructors using POMS and found that training had a detrimental effect on overall mood. Specifically, significant main effects of timepoint were observed on total mood disturbance, vigor–activity, confusion–bewilderment, and fatigue. Perroni et al. [42] examined POMS in firefighters during simulated firefighting activities and observed no differences between pre- and post-interventions.

#### 3.4.5. Subjective Stress Perception

Three studies examined subjective stress perception on a 1–100 scale [21,28,54]. In a study that analyzed the psychophysiological response of soldiers undergoing a special operations selection course, subjective stress perception increased significantly from baseline to immediately following the selection course [28]. Additionally, significant increases in SSP were observed in aircrews following underwater evacuation training [54].

#### 3.4.6. Other Subjective Outcome Measures

A variety of studies include subjective outcome measures that were only mentioned once. These include the Kessler-10 [23], Depression Anxiety Stress Scale (DASS) [23], Life Engagement Test [28], Coping Flexibility Scale [28], Acceptance and Action Questionnaire (AAQ-II) [28], Visual Analogue Scale (VAS) [28], Effort–Reward Imbalance Questionnaire (ERIQ) [32], Connor–Davidson Resilience Scale (CD-RISC) [35], perceptions of thermal sensations and respiratory distress [43], a general fatigue questionnaire [48], Clinician Administered Dissociative States Scale (CADSS) [16,53], Impact of Events Scale-Revised (IES-R) [16], Multidimensional Fatigue Inventory (MFI) [53], and the NASA Task Load Index (NASA-TLX) [56].

Significant changes were observed in each of the following: DASS [23], VAS [28], ERIQ [32], perceptions of thermal sensations and respiratory distress [43], and IES-R [16]. The DASS is an extensive questionnaire measuring psychological distress and assesses three dimensions of perceived depression, anxiety, and stress. Chester et al. [23] utilized this assessment in Royal Australian Air Force Personnel during military environmental survival training (EST) and observed significant increases in all three subscales from pre- to post-EST.

The VAS is used to measure global motivation loss and affect and is based on eight unipolar VAS ratings including alertness, sleepiness, motivation loss, weariness, happiness, sadness, calmness, and tension. Hormeno-Holgado et al. [28] analyzed the psychophysiological response of soldiers undergoing a special operations selection course and found significant negative changes in all VAS ratings except sleepiness.

The ERIQ assesses effort and reward as well as provides an effort to reward ratio. Izawa et al. [32] examined effort–reward imbalance and its relation to inflammatory markers in police officers working 24 h shifts. Significant effects of effort and effort–reward ratio on cortisol secretion were detected, meaning higher effort scores and effort–reward ratios were associated with lower cortisol levels.

Petruzzello et al. [43] examined perceptions of thermal sensations (TS) and respiratory distress (RD) in career and volunteer firefighters and observed significant increases in both TS and RD from pre- to post-live-fire training drills.

The IES-R is a 22-item self-report questionnaire that assesses current subjective distress for any specific live event and is broken down into three subscales corresponding to PTSD symptoms: avoidance (IES-Avoid), intrusion (IES-Intrusion), and hyperarousal (IES-Arousal) [16]. Taylor et al. examined endocrine reactivity and psychological impact during stressful military training and observed significant positive associations between IES-Avoid and IES-Arousal and cortisol concentrations during stressful military captivity. Additionally, dissociative symptoms were significantly positively associated with IES-Avoid and IES-Intrusion.

### 3.5. Summation

Supplemental Table S2 provides a summation of these measures and the direction of their findings (i.e., increase, decrease, or no change). Overall, the most common objective outcome measures of stress were HR (n = 24), Cortisol (n = 16) and body temperature (n = 13), while the most common subjective outcome measure of stress were RPE (n = 14) and STAI (n = 11). For both objective and subjective measures, there were multiple outcome measures that were used only once. In over half of the studies (n = 22), both an objective and subjective outcome measure was used. These results suggest that the utilization of both an objective and subjective outcome measure may provide the best utility when profiling occupational task stress.

## 4. Discussion

The primary aim of this review was to capture and critically appraise research investigating subjective and objective measures of physiological stress in tactical populations. Forty-two studies were included and were generally of high-quality due to the study design of majority of the articles (cohort, quasi-experimental, and case-control). Of the multiple objective and subjective stress measures that were assessed, emerging themes were found where outcome measures either increased, decreased, or produced variable responses depending on the task or event. These emerging themes are discussed in greater detail below.

### 4.1. Objective Measures

#### 4.1.1. Measures Found to Typically Increase in Response to Stress

Heart rate (beats per minute: bpm) was the most frequently examined measure during operational tasks, with over half of the articles including it as an objective measure [15,20,24,26,28,29,30,33,34,36,37,43,44,45,46,47,48,49,50,54,55,56,57,62,63]. It is well known that HR increases in response to an acute, stressful event Reflecting part of the body’s “fight or flight” response [15]. An increase in HR as measured by bpm was observed in all studies that examined HR following a task or simulated operation. Increases varied depending on the task being performed. In a singular event, such as a parachute jump, HR increased in novel and experienced warfighters between 12–15 bpm on average [24]. In a prolonged event, such as military survival training, HR increased up to 81% as compared to baseline measures [15]. Marcel-Millet et al. examined heart rate recovery (HRR), the time it takes for HR to return to normal following cessation of a task or event in firefighters during multiple rescue interventions and a maximal intermittent fitness test [36]. HR was taken at 60 s and 300 s following each intervention. HRR was significantly lower in all firefighting rescue interventions as compared to the maximal incremental fitness test, showing the rescue interventions led to a greater disturbance in parasympathetic reactivation. The cardiovascular strain imposed on tactical personnel can be of concern, as this may lead to an increased risk of cardiovascular disease (CVD) [13,14].

In stressful events that result in cardiovascular strain, there is often some degree of thermoregulatory strain as well [30]. This combination of cardiovascular and thermoregulatory strain can predispose individuals to heat-related exhaustion [30,55,57], where blood is redistributed away from the central circulatory system in attempt to cool the extremities, inhibiting the body’s ability to regulate core temperature under physiological stress. Significant increases in body temperature, whether it be skin or core temp, were observed in all studies where firefighters were participating in real or simulated firefighting tasks [29,30,34,43,55,57]. When the body’s ability to autoregulate core temperature is diminished, heat tolerance time is decreased, cardiac output decreases while HR increases, and aerobic power and muscular endurance are reduced significantly [66,67]. Additionally, perception of exertion is increased, while attention, vigilance, and short-term memory are decreased [66,68]. Each of these physiological changes, resulting from heat stress, reduce the ability of the tactical operator to perform their job optimally. When both cognitive and physical performance begin to diminish due to poor thermoregulation, the risk of making mistakes on the job, the risk of injury to self or others, and the probability of a heat-related illness all increase [69].

Hunt et al. [30] utilized an adapted Physiological Strain Index (aPSI) that provides a rating of strain based on core temperature, skin temperature, and heart rate. The aPSI produced significantly high ratings of physiological strain in firefighters during simulated fire training scenarios. While the volume of evidence is limited because very few studies have utilized the aPSI, this tool could provide useful information in tactical personnel. In addition to observations of heart rate and core temperatures, Horn et al. [29] sought to examine the hemostatic response in firefighters and instructors during various live-fire training environments and observed significant increases in platelet count and platelet closure time. Increases in platelet number and aggregation are associated with unstable chest pain or discomfort and heart attacks [70,71]. Assessment of hemostatic function in combination with HR and core temperature can be useful to help examine physiological strain experienced in environments where individuals encounter high levels of physical exertion and are exposed to high heat in long durations, such as live-fire training activities or simulated military operations in extreme temperatures.

Additional physiological measures that have been seen to increase during an acute, stressful event are blood lactate and creatine kinase (CK). Blood lactate increases as a result of the body’s neuroendocrine response to convert lactate to glucose to use as an energy substrate via the Cori cycle once other sources begin to deplete [46]. All studies that investigated blood lactate concentration in tactical personnel during an event observed significant increases [21,24,37,45,46,47,49,50,62,63], with the exception of findings from Vartanian et al. [53]. This could be due to researchers examining stress in instructors carrying out military survival training as opposed to the individuals who are going through the survival training, where the demands placed on the instructors may not be as high. Creatine kinase has been proposed as an indirect indicator of muscle damage and has been used to assess training intensity and as a marker of over training [65]. Due to the intense nature of military training and selection courses, muscle damage and effects of over training are often observed in correlation with the increase in CK levels [23,40]. Accordingly, all studies that examined CK as an outcome measure observed increases [23,24,27,40]. The increase in CK levels observed by Clemente Suarez et al. [24], although not significant, provide information showing that acute stressful events such as a parachute jump can still result in an increase in CK.

#### 4.1.2. Measures Found to Typically Decrease in Response to Stress

Measures which typically decreased in response to stress, included SpO_2_, CFFT, hydration levels, urine pH and urine glucose. Blood oxygen saturation (SpO_2_) during strenuous or physically demanding activities initially drops as the working muscles receive more oxygen [72]. The body adapts to lower levels of oxygen in the blood by increasing breathing rate [72]. In the period following an event, SpO_2_ levels should return to normal. In most studies that examined SpO_2_ during military combat operations, decreases were observed, with the exception of findings from Bustamante-Sanchez et al. [21] and Hormeno-Holgado et al. [28]. Bustamante-Sanchez et al. [21] observed no change in SpO_2_ from pre- to post-helicopter flight in the Spanish Air Force. While there is a great mechanical load in flight due to vibrations, G forces, etc., the relative physical demand on the muscles in flight may be lower versus ground combat operations, resulting in little to no change in SpO_2_. In a study monitoring soldiers in a Special Operations Course, Hormeno-Holgado et al. [28] observed a significant increase in SpO_2_ from baseline to immediately following completion of the 4-day course. Although the increase was found to be significant, SpO_2_ values only increased slightly from 98.0 ± 1.1 to 98.7 ± 0.7. The timing of data collection for the Hormeno-Holgado et al. [28] study could have influenced the results, with the candidates having completed the intense selection course and entering recovery mode. Thus, while the volume of evidence favors a decrease in SpO_2_, the evidence on increases requires further investigation as this could inform stress recovery protocols.

The Critical Flicker Fusion Threshold (CFFT) has been used in the research as a way to measure cortical arousal. Decreases in cortical arousal are often observed in combat scenarios where individuals are placed in stressful situations with increased physical demands [9,62]. Decreases have also been observed in helicopter flights [21], parachute jumps [24,45], and shift schedules in firefighters [48]. Although not significant, decreases in values were observed in all studies utilizing the CFFT [21,24,28,45,46,47,48,49,50,54,62,63]. This decrease in cortical arousal can be associated with CNS fatigue and an impairment in executive functions required for information-processing and decision making [28], each of which are critical for the tasks and duties tactical operators must carry out.

In soldiers going through a special operations selection course, urine samples were collected to analyze dehydration [28], with levels decreasing significantly following the last phase of the selection course. Dehydration is commonly seen in courses lasting longer than a few days where physical activity is high, leading to increased sweat rate, and fluid intake is low [73]. In addition to assessing urine color, researchers utilized the urine Combur-test and observed significant negative changes in urine pH and glucose [28]. This could be a result of insufficient recovery from lack of sleep, a caloric deficit, and exertional fatigue [74,75], all of which negatively affect the stress response.

#### 4.1.3. Measures Found to Have Variable Responses to Stress

Several outcome measures produced variable responses to stress including all serum and salivary hormones as well as HRV. Multiple serum and salivary hormones have been used as markers of physiological stress. While the responses from individual hormones varied from others, specific ones did follow trends of increasing or decreasing. Hormones examined in this review that presented with significant increases following strenuous tasks performed in operational environments include cortisol, DHEA, SHBG, epinephrine (EPI), norepinephrine (NE), dopamine, StFR, IL-6, C-reactive protein (CRP), hepcidin, alpha-amylase, and neuropeptide Y (NPY). Cortisol was the most examined hormone included in this review, with sixteen studies using it as an objective marker of stress. In all studies that examined cortisol as a measure of physiological stress, increased levels were observed [15,16,20,22,24,27,32,35,39,41,42,44,51,52,53,64]. Cortisol is a catabolic hormone and increases under prolonged periods of stress as a result of changes to the HPA axis, resulting in a decrease of the body’s regulatory mechanisms for survival [8]. Conditions tactical personnel are often placed in, such as those in Survival, Evasion, Resistance, and Escape (SERE) school [15], stressful captivity [16], military selection courses [27], or fighting fires [42] require increased physical demands under duress for prolonged periods. These high-stress conditions trigger a catabolic response from increased levels of cortisol, leading to increased fatigue and poor performance during job tasks [76].

Significant increases in DHEA were observed in stressful captivity in military survival training in soldiers [16] and in instructors [53]. Following survival training in instructors, DHEA levels remained elevated in the 3-day recovery period that followed [53]. Levels of DHEA in the plasma typically persist longer, taking more time to return to normal following exposure to stress [77]. It is possible that DHEA may produce a buffer mechanism during stress, in which over time, baseline levels of DHEA begin to increase after repeated stressful exposures [77]. Morgan and colleagues observed soldiers enrolled in the military Combat Diver Qualification Course (CDQC) who exhibited higher levels of stress-induced DHEA also exhibited fewer stress-induced symptoms of disassociation, theorizing that higher baseline levels may protect against significant stress-induced deficits [77]. In multiple studies examining prolonged military field training (MFT), significant increases in SHBG were observed [27,41,51,52]. Similar changes in hormonal profiles have been observed in studies examining common aspects of MFT, such as sleep and calorie restriction combined with intense physical activity [78,79].

The body’s initial neural response to an acute stressor, mediated by the sympathetic nervous system, is a release of EPI, NE, and dopamine [15]. This is a critical component to the body’s fight or flight response leading to increased arousal and blood flow to the brain, vasodilation in muscles, peripheral vasoconstriction, and an increased heart rate, which are adaptive functions for survival [15]. Significant increases were observed in plasma EPI and NE in soldiers in SERE school [15] and during military survival training [64]. A significant increase in dopamine was observed in soldiers undergoing military survival training [64].

Inflammatory biomarkers, such as IL-6 and C-reactive protein, and iron levels were observed in multiple studies. Serum hepcidin and IL-6 were examined during a 7-day winter military training exercise where researchers observed significant increases, raising the possibility that repeated exposure to strenuous tasks may degrade iron status [38]. Soluble transferrin receptor (sTfR), the gold-standard indicator for iron status, was unchanged in this same study [38], but was seen to increase significantly in soldiers during SERE school [15]. C-Reactive Protein (CRP) was assessed during one week of a Special Forces annual selection course as a measure of systemic inflammation where significant increases were observed from baseline to the end of the week [27]. Similar results were found in male soldiers at the end of an Army Ranger course, in whom inflammatory markers such as CRP and IL-6 were markedly increased [80], suggesting the physiological stress of arduous military training is sufficient enough to result in high levels of systemic inflammation.

Perroni et al. [42] observed significant increases in salivary alpha-amylase during a simulated fire-fighting intervention in male firefighters. These results presented with a typical alpha-amylase response in line with the response of the sympathetic nervous system (SNS) to stress [81]. Nueropeptide Y (NPY) is also released as an SNS response to stress and was found to increase significantly in SERE students [15]. Previous studies have examined NPY in stressful circumstances and have seen similar increases associated with both superior military performance and reduced psychological function [76].

Hormones that presented with significant decreases include testosterone, IGF-1, TNF-alpha, leptin, T3 and T4. Testosterone is often observed in studies examining high-stress scenarios. Significant decreases in serum and salivary testosterone were observed in all studies using TES as a physiological marker [15,27,39,41,51,52,53,64]. Testosterone is an anabolic hormone that aids in muscle protein synthesis and supports muscle growth and strength [82]. A decrease in serum testosterone is an indicator of reduced HPA activity leading to blunted protein synthesis and muscle-building properties [82]. Low levels of testosterone are often a result of energy and sleep deprivation, caloric restriction, and increased physical and psychological stress [15,27,83], all of which are a consequence of prolonged military field and survival training [15,27,39,41,52,53,64]. Significant reductions in serum IGF-1 were observed in military field training [40,41], U.S Army Ranger training [39], and an 11-week paratrooper training course [52]. IGF-1 is sensitive to changes in energy intake, specifically in dietary protein, where inadequate intake will result in significant decreases in IGF-1 [39,84]. Additionally, a decline in IGF-1 is an indication of a decreased ability of the body to deposit protein for muscle tissue growth [39] making it more difficult for individuals to recover from the intense physical demands experienced during training that lasts for days at a time.

Serum TNF-alpha was investigated in subjects of the Finnish Army going through prolonged military field training (MFT), whose levels initially increased but were followed by a significant decrease as time went on [40]. Levels then started to return to baseline following four days of recovery. Previous research on TNF-alpha in extreme settings and environments has not presented with consistent findings, but increases have been suggested to be a result of inflammation caused by muscle damage [85]. In the same study, researchers examined serum leptin levels and observed significant decreases from baseline to midway- and post-timepoints but recovered in the days following. Leptin concentrations were associated negatively with CK levels, showing changes in leptin may also be indirect indicators of muscle damage [40]. Prior studies have shown that energy deficit, as experienced in prolonged MFT, could be an influencing factor on decreases in leptin concentration [86]. The T3:T4 ratio decreased significantly in a study examining Special Forces soldiers going through a selection course [27], implying a reduced conversion of T3 from T4. This decrease is expected, as seen in previous research with strenuous physical activity where individuals were sleep-deprived and calorie-restricted [84].

Heart rate variability (HRV) is often used when examining stress and recovery and was reported in 11 studies. HRV is an expression of neurocardiac function generated by interactions between the heart and brain and the dynamic processes of the autonomic nervous system [87]. Varying results were observed in each of the HRV metrics examined in the studies utilizing HRV as an objective outcome measure. RMSSD values and HF values are typically strongly correlated, as both are influenced by the variance in vagal tone from parasympathetic activity [87]. In stressful environments, sympathetic activity increases. This results in a decrease in RMSSD and HF values, indicating a stressor may have been experienced that the individual cannot cope with [33,36,45,47,49]. Only two studies observed increases in RMSSD and HF values [54,63]. Increases observed in a study by Vicente-Rodriquez et al. [54] could be a result of underwater training where individuals experience a slowed HR from breath holding, a result of the mammalian dive response [88]. In addition to metrics of RMSSD and HF values, studies that observed LF values resulted in both increases [45,47,49,63] and decreases [37,54], although disagreement between studies in LF values is not unexpected because of the multifactorial influences on the LF domain [87].

### 4.2. Subjective Measures

Much like objective measures, many subjective measures were found only to increase when stress was applied. These measures include rating of perceived exertion (RPE), subjective stress perception (SSP), the Depression Anxiety Stress Scale (DASS), and perceptions of thermal sensations (TS) and respiratory distress (RD). The remaining subjective measures produced variable responses under stress, including STAI, CSAI-2R, POMS, and VAS. Each of these will be discussed in detail below.

#### 4.2.1. Measures Found to Typically Increase in Response to Stress

An RPE scale was the most commonly observed subjective measure in tactical personnel during stressful events with just over half of the studies that examined subjective measures utilizing it [21,26,28,36,37,40,43,45,46,49,54,56,62,63]. All but two studies that examined RPE observed an increase in ratings of perceived exertion following a tactical operation. Marins et al. [37] examined the effects of personal protective equipment (PPE) on law enforcement officers during an occupational physical ability test and observed little to no change in RPE between groups with and without PPE. This could be due to tasks being performed under a fixed pace for both conditions, leading to the possibility of officers not overexerting themselves during the test. In contradiction to these findings, other studies have observed increases in RPE under loaded conditions [89,90]. In a study examining physiological and perceptual responses to live-fire training drills, researchers observed RPE measures, but only at the conclusion of the drill [43]. With RPE not being recorded prior to the start of the event, no changes were observed. Previous research has discussed over- and underestimations of RPE, depending on the intensity and duration of the event or task being performed [62]. Ratings of Perceived Exertion are commonly examined in correlation with blood lactate concentration to assess incongruences in subjective and objective reporting [91]. Incongruences in subjective reporting and objective physiological workload measures can be a result high-stress situations having a negative impact on superior cognitive processes that include perception, attention, executive function, and memory [92]. This should be taken into consideration when using RPE as a measure of stress in tactical personnel.

Subjective stress perception (SSP) was examined in three studies [21,28,54], all resulting in an increase in stress perception. Participants rated perceived stress to a task or event on a scale of 1 to 100, with 1 being no stress at all and 100 being highly stressed. Similar to RPE, SSP can be examined in correlation with objective measures. Vicente-Rodriquez et al. [54] observed significant changes in HR and HRV values following underwater evacuation training in line with increases in SSP, indicating similar increases in sympathetic nervous system modulation.

The Depression Anxiety Stress Scale (DASS) utilized in Air Force personnel undergoing military environmental survival training (EST) [23] resulted in significant increases in all three dimensions of perceived depression, anxiety, and stress. Although the volume of evidence in tactical personnel from previous studies using the DASS scale is small, these results further support the use of self-reported subjective data to observe psychological distress during combat-like scenarios.

Perceptions of thermal sensations (TS) and respiratory distress (RD) were only reported in one study investigating firefighters during live-fire training drills [43]. Findings from this study are consistent with previous findings in firefighters [93] in whom significant increases were observed from pre- to post-measures. While no correlations were examined between subjective and objective measures in the study examining TS and RD [43], HR and core temp both resulted in significant increases as well, suggesting these measures could be observed in accordance with the other for further examination of perceived and actual stress in firefighters.

#### 4.2.2. Measures Found to Have Variable Responses to Stress

The use of the State–Trait Anxiety Inventory (STAI), a 20-item self-report scale that assess two parts of anxiety: an individuals’ temporary condition of state anxiety and their predisposition to trait anxiety [42], resulted in variable responses depending on the task and environment in observation. STAI was the second most commonly observed subjective measure with just over a third of the studies examining subjective data utilizing it [21,28,31,42,43,44,45,46,47,49,63], although only four of the studies observed significant findings [28,31,44,63]. In studies that examined STAI in flight, one found no change in state anxiety from pre to post flight [21], while the other observed significantly higher anxiety levels pre-flight compared to non-flight and post-flight [31]. Pre-flight state anxiety could be a result of performance anxiety experienced before performing an important or challenging task [31]. Hormeno-Holgado et al. [28] observed a significant decrease in state anxiety in soldiers undergoing a special operations selection course, while other studies observed no change or an increase in state anxiety during simulated combat operations [45,46,47,49,63] or firefighting interventions [42]. With the special operations selection course lasting longer than the single event simulated operations, the increase in state anxiety observed in the simulated operations could be a result of acute hyperreactivity of the autonomic nervous system in a stressful scenario [45]. State-anxiety levels may decrease following completion of the selection course as the individual begins to feel a sense of accomplishment from completing a long and challenging course [28].

Much like the STAI, The Competitive State Anxiety Inventory (CSAI-2R) and the Profile of Mood States (POMS) also produced variable responses in each of the studies utilizing them. The CSAI-2R assessment of somatic anxiety (SA), cognitive anxiety (CA) and self-confidence (SC) has been validated [94] and used in previous military research [21,63]. Increases in CA and SA indicate a higher level of anxiety, while increases in SC indicate higher levels of self-confidence [21]. Inconsistent findings in self-reported anxiety were observed in all studies investigating the CSAI-2R [21,24,45,46,47,49,63]. For example, during combat simulations, two studies observed an increase in SA [46,49] while others observed a decrease in SA [47,63]. In those same studies, two observed an increase in CA [46,49], while the others observed decreases in CA [47,63]. In contradiction to reports of anxiety, self-confidence resulted in consistent findings, with increases being observed in all studies, although results were not significant. This could be a result of feelings of increased confidence after completion of a challenging or demanding task. Varying results from the POMS could be a reflection of the different environments this assessment was being observed in. One study examined POMS during a single-event simulated firefighting activity and observed no changes [42], while the others assessed mood states during longer duration, stressful training in arduous environments [15,23,53] and observed significant negative changes. Specifically, Vartanian et al. [53] investigated stress in instructors of the military survival captivity training and found that training had a significant, detrimental effect on overall mood, vigor–activity, confusion–bewilderment, and fatigue, similar to responses observed in students undergoing stressful military training [15,23]. Previous studies have observed similar mood deteriorations in warfighters engaged in field training activities [5,95,96].

The Visual Analog Scale (VAS) is used to assess global motivation loss and affect [97]. There are four VAS ratings primarily concerned with motivation loss (alertness, sleepiness, motivation loss, and weariness), and four ratings concerned with the affective state (happiness, sadness, calmness, and tension), resulting in two outcome measures: global motivation loss (GV) and global affect (GA). Global motivation loss and global affect ranged in value from zero to one hundred. Hormeno-Holgado et al. [28] monitored the psychophysiological response of soldiers throughout a special operations selection course and utilized the VAS scale to assess affect and vigor. Immediately following completion of the course, a significant increase was observed in motivation loss, weariness, happiness, and calmness, while a significant decrease was observed in alertness, sadness, and tension. A small decrease was observed in sleepiness ratings, although not significant. Overall, GV decreased significantly, and GA increased significantly. These results are in line with previous findings in fatiguing, stressful environments where motivation loss and weariness increased, and alertness, sadness, and tension immediately decreased following completion of the training [74]. This could be explained by soldiers’ sense of accomplishment after finishing a course, such as a special operations selection course.

### 4.3. Objective and Subjective Measures—Was Significance Found in Both?

While more than half of the included studies (n = 22) utilized both objective and subjective measures of stress [15,16,21,23,24,28,31,32,35,40,42,43,44,45,46,47,48,49,53,54,56,63], few examined each of them in relation to each another to determine if interactions exist in responses between measures. Vartanian et al. [53] addresses the issue of how one’s psychological make up may affect an individual’s response to stress, suggesting a need to examine the relationships between psychological and physiological measures. A significant correlation was observed in salivary alpha-amylase (sA-A) and state anxiety scores from the STAI assessment at individual timepoints in air self-defense pilots [31], suggesting sA-A may be a useful indicator of acute psychological stress. In contradiction to these findings, sA-A and salivary cortisol were examined in correlation with STAI and POMS scores in firefighters, where low, nonsignificant correlations were observed [42]. These results could indicate salivary measures changing in response to the physical demands of simulated firefighting tasks rather than psychological demands. Researchers observed little to no change in psychological measures from pre- to post-intervention, suggesting firefighters did not perceive the training environment as dangerous or threatening [42].

Izawa et al. [32] examined cortisol secretion and inflammatory activity in relation to effort–reward imbalance in police officers working 24 h shifts. Work environments that require high amounts of effort with low levels of reward are particularly stressful and could lead to poor health conditions from chronic elevation of HPA activity [98]. Researchers found that higher effort scores and effort–reward ratios were associated with lower cortisol levels [32], indicating there could be dysregulation of the HPA axis with a blunted cortisol response or a decreased awakening response of cortisol release—a result of higher negative feedback sensitivity [98]. These results could contribute to the previously mentioned stress-related diseases experienced in police officers, such as cardiovascular disease [13,14].

Cortisol secretion, as well as DHEA, were examined in correlation with the Impact of Events Scale (IES-R) in Navy personnel undergoing stressful captivity training [16]. Results showed that hormonal responses of both cortisol and DHEA during stressful captivity may influence psychological impact, although in differing ways. IES-Avoid and IES-Intrusion were significantly, positively associated with cortisol concentrations during stressful captivity, while IES-Arousal was significantly, inversely associated with percent change in the DHEA-cortisol ratio. Heightened levels of cortisol intensified avoidance mechanisms and intrusive thoughts, while the increased DHEA-cortisol ratio reduced the physiological arousal that typically happens as a result of stressful events [16]. These physiological responses further the notion that physical stressors may have a negative effect on cognitive processes, which are critical to many of the duties tactical operators must carry out [76,77].

### 4.4. Review Limitations

There are some limitations to this review. Across all students, there was little to no consistency in the subjective or objective measures employed. While some common objective measures (both subjective and objective) were identified, there was still a large variability in the range of tasks and environments used to induce stress. Thus, the high variety of outcome measures used and the diverse variables used to induce stress (including both type and duration) make establishing a volume of evidence challenging. Considering this, tactical tasks will vary and be diverse based on the tactical unit’s role and scope. Thus, while consistency in methodological approaches to employ stress is lacking it is realistic. Hence, the identification of the most common objective and subjective outcome measures, as identified in this review, can aid in at least establishing common measures through which to inform levels of stress associated with tactical tasks and occupations.

## 5. Conclusions

Based on key findings, a wide variety of objective and subjective outcome measures are used to determine the amount of occupational stress experienced in extreme environments and tasks tactical personnel often find themselves in. The most commonly observed objective outcome measures included HR, cortisol, and body temperature, while the most commonly observed subjective outcome measures were RPE and STAI. Interestingly, over 50% of studies employed both an objective and a subjective outcome measure, although in combination, these results may have differed and not supported each other. Future research should seek to employ these common outcome measures across a range of tactical occupational tasks in order to develop a volume of evidence based on similar outcome measures. Furthermore, many of the studies observed acute, shorter-term, scenarios, and as such, resiliency to repeated exposures over long periods of time is lacking and should be examined for deeper insights into overall health and performance in tactical professionals.

## Figures and Tables

**Table 1:** Keywords used to search relevant databases.

Database	Filters Applied	Population		Target Variable		Exclusion Terms
PubMed	2005–2020, human species, English language, Adult 19+ years	‘Military’ OR ‘Military Personnel’ [Mesh] OR ‘Tactical Personnel’ OR ‘Infantry’ OR ‘Navy’ OR ‘Naval’ OR ‘Air Force’ OR ‘Army’ OR ‘Armed Forces’ OR ‘Special Forces’ OR ‘Special Operations’ OR ‘Warfighter’ OR ‘Police’ [Mesh] OR ‘Law Enforcement’ OR ‘Firefighters’ [Mesh] OR ‘Fireman’ OR ‘Emergency Responders’ [Mesh]	AND	‘Stress’ OR ‘Exercise’ OR ‘Endocrine’ OR ‘Immune’ OR ‘Hormone’ OR ‘Physiological’ OR ‘Physical Exertion’ OR ‘Physical Demands’ OR ‘Operational Demands’ OR ‘Perceived Demands’ OR ‘Fatigue’ OR ‘Train’ OR ‘Strenuous’ OR ‘Threat’ OR ‘Trauma’ OR ‘Strain’ OR ‘Cortisol’ OR ‘Neuroendocrine’ OR ‘Readiness’ OR ‘Resilience’ OR ‘Hardiness’ OR ‘Exhaustion’ OR ‘Biomarker’	NOT	‘Disorder’ OR ‘Post Traumatic Stress’
Embase	2005–2020, adult (18–64 years), human	‘Military’ OR ‘Military Personnel’/exp OR ‘Tactical Personnel’ OR ‘Infantry’ OR ‘Navy’ OR ‘Naval’ OR ‘Air Force’ OR ‘Army’ OR ‘Armed Forces’ OR ‘Special Forces’ OR ‘Special Operations’ OR ‘Warfighter’ OR ‘Police’/exp OR ‘Law Enforcement’ OR ‘Firefighters’/exp OR ‘Fireman’ OR ‘Emergency Responders’/exp	AND	‘Stress’ OR ‘Exercise’ OR ‘Endocrine’ OR ‘Immune’ OR ‘Hormone’ OR ‘Physiological’ OR ‘Physical Exertion’ OR ‘Physical Demands’ OR ‘Operational Demands’ OR ‘Perceived Demands’ OR ‘Fatigue’ OR ‘Train’ OR ‘Strenuous’ OR ‘Threat’ OR ‘Trauma’ OR ‘Strain’ OR ‘Cortisol’ OR ‘Neuroendocrine’ OR ‘Readiness’ OR ‘Resilience’ OR ‘Hardiness’ OR ‘Exhaustion’ OR ‘Biomarker’	NOT	‘Disorder’ OR ‘Post Traumatic Stress’
Ebsco (CINAHL and SPORTDiscus)	2005–2020, all adult, English language	‘Military’ OR (MH ‘Military Personnel+’) OR ‘Tactical Personnel’ OR ‘Infantry’ OR ‘Navy’ OR ‘Naval’ OR ‘Air Force’ OR ‘Army’ OR ‘Armed Forces’ OR ‘Special Forces’ OR ‘Special Operations’ OR ‘Warfighter’ OR (MH ‘Police+’) OR ‘Law Enforcement’ OR (MH ‘Firefighters+’) OR ‘Fireman’ OR (MH ‘Emergency Responders+’)	AND	‘Stress’ OR ‘Exercise’ OR ‘Endocrine’ OR ‘Immune’ OR ‘Hormone’ OR ‘Physiological’ OR ‘Physical Exertion’ OR ‘Physical Demands’ OR ‘Operational Demands’ OR ‘Perceived Demands’ OR ‘Fatigue’ OR ‘Train’ OR ‘Strenuous’ OR ‘Threat’ OR ‘Trauma’ OR ‘Strain’ OR ‘Cortisol’ OR ‘Neuroendocrine’ OR ‘Readiness’ OR ‘Resilience’ OR ‘Hardiness’ OR ‘Exhaustion’ OR ‘Biomarker’	NOT	‘Disorder’ OR ‘Post traumatic stress’

**Table 2:** Participants and Demographics.

Author	Country	Participants/Demographics
Backe et al., 2009 [20]	Germany	Urban ambulance personnel
		n = 24 (19 male, 5 female)
		Age: 28 yrs (range: 20–43 yrs)
		Average time in service: 7 yrs (range: 1–25 yrs)
Bustamante-Sanchez et al., 2020 [21]	Spain	Spanish Army aircrew members
		n = 12 (male)
		Age: 30.4 ± 4.4 yrs
		Height: 173.8 ± 2.3 cm
		BMI: 23.9 ± 2.4 kg/m^2^
		Years of experience: 11.2 ± 3.7
Charles et al., 2016 [22]	USA	Police officers
		n = 319 (248 male, 71 female)
		Day shift (n = 134)
		Afternoon shift (n = 106)
		Night shift (n = 79)
		Age: 42.8 ± 7.7 yrs
		Waist circumference: 95.5 ± 14.0 cm
Chester et al., 2013 [23]	Australia	RAAF Personnel
		n = 14 (9 male, 5 female)
Clemente-Suarez et al., 2016 [24]	Spain	Soldiers of the Spanish Army
		n = 40
		Novel (n = 17, mean age: 23.2 ± 2.7 yrs; BMI: 23.7, 6 parachute jumps)
		Experienced (n = 23, mean age: 35.5 ± 5.2 yrs, BMI: 76.0, 76.0 ± 10.8 parachute jumps)
Clemente-Suarez et al., 2017 [25]	Spain	Soldiers of the Spanish Army and State Security Corps
		n = 20
		Age: 34.5 ± 4.2 yrs
		Height: 176.4 ± 8.4 cm
		Weight: 74.6 ± 8.7 kg
Gomez-Oliva et al., 2019 [26]	Spain	Soldiers of the Spanish Armed Forces
		n = 20
		Age: 33.3 ± 5.4 yrs
Hamarsland et al., 2018 [27]	Norway	Soldiers participating in selection course for Norwegian Special Forces
		n = 15
		Age: 23 ± 4 yrs
		Height: 1.81 ± 0.06 m
		Weight: 78 ± 7 kg
Hormeno-Holgado et al., 2018 [28]	Spain	Professional soldiers from Special Operations Unit
		n = 46
		Average experience: 29.1 ± 59.3 months
		Age: 25.1 ± 5.0 yrs
		Height: 1.8 ± 0.1 m
		Weight: 76.8 ± 7.9 kg
		BMI: 24.4 ± 2.5 kg/m^2^
Horn et al., 2019 [29]	USA	Firefighters (n = 24, 22 males, 2 females)
		Age: 40.4 ± 1.8 yrs
		Height: 1.81 ± 0.01 m
		BMI: 27.5 ± 0.9 kg/m^2^
		Average experience: 16.6 ± 1.6 yrs
		Fire instructors (n = 10, 9 males, 1 female)
		Age: 34.7 ± 2.2 yrs
		Height: 1.78 ± 0.03 m
		BMI: 27.2 ± 1.3 kg/m^2^
Hunt et al., 2019 [30]	Australia	Male firefighters serving in the Australian Defence Force
		n = 9
		Age: 29.2 ± 2.3 yrs
		Height: 177.9 ± 7.7 cm
		Weight: 91.3 ± 8.6 kg
Iizuka et al., 2012 [31]	Japan	Japan Air Self-Defense Force pilots
		n = 9
		Age: 36.0 ± 6.0 yrs
Izawa et al., 2016 [32]	Japan	Male police officers
		n = 142
		Age: 43.0 ± 9.1 yrs
		BMI: 24.3 ± 2.6 kg/m^2^
Kaikkonen et al., 2017 [33]	Finland	Firefighters
		n = 21
		Age: 38.0 ± 7.0 yrs
		Height: 178.0 ± 70 cm
		BMI: 25.0 ± 2.0 kg/m^2^
Kesler et al., 2017 [34]	USA	Firefighters
		n = 30 (29 males, 1 female)
		Age: 30.4 ± 1.5 yrs
		Height: 1.82 ± 0.01 m
		BMI: 27.4 ± 0.7 kg/m^2^
Ledford et al., 2020 [35]	USA	Navy SEAL candidates in BUD/S
		n = 116
		Age range: 17–35
Lieberman et al., 2016 [15]	USA	Male SERE school students
		n = 60 (15 officers, 45 enlisted personnel)
		Age: 26.9 ± 0.4 yrs
		Weight: 85.4 ± 1.0 kg
		Average years of experience: 4.4 ± 2.7
Marcel-Millet et al., 2018 [36]	France	Firefighters
		n = 34
		Males (n = 28)
		Age: 37 ± 4 yrs
		Height: 179 ± 6 cm
		BMI: 24 ± 2 kg/m^2^
		Females (n = 6)
		Age: 29 ± 3 yrs
		Height: 171 ± 4 cm
		BMI: 22 ± 1 kg/m^2^
Marins et al., 2018 [37]	USA	Male Federal Highway Police
		n = 13
		Age: 36.8 ± 3.7 yrs
		Height: 180 ± 5.6 cm
		Weight: 89.0 ± 10.7 kg
		BMI: 27.5 ± 2.9 kg/m^2^
McClung et al., 2013 [38]	USA	Male Norwegian Soldiers
		n = 21
		Age: 20 ± 1 yrs
		Height: 182 ± 7 cm
		Weight: 82+9 kg
		BMI: 25 ± 2 kg/m^2^
Nindl et al., 2007 [39]	USA	Male soldiers of the U.S Army
		n = 50
		Age: 24.6 ± 4.1 yrs
		Height: 176.1 ± 7.8 cm
		Weight: 78.4 ± 8.7 kg
		BMI: 25.6 ± 4.2 kg/m^2^
Ojanen et al., 2018 [40]	Finland	Members of the Finnish Army
		n = 49
		Age: 20 ± 1 yrs
		Height: 179 ± 6 cm
		Weight: 73.5 ± 8.7 kg
		Body fat: 12.6 ± 5.0%
Ojanen et al., 2018 [41]	Finland	Male Finnish Army conscripts
		n = 49
		Age range: 19–22 yrs
		Height: 178.5 ± 6.4 cm
		Weight: 73.5 ± 8.7 kg
		Body fat: 12.6 ± 5.0%
Perroni et al., 2009 [42]	Italy	Male Italian firefighters
		n = 20
		Age: 32 ± 1 yrs
		Height: 1.77 ± 0.06 m
		Weight: 77.2 ± 8.6 kg
		BMI: 24.7 ± 2.1 kg/m^2^
Petruzzello et al., 2014 [43]	USA	Male career and volunteer firefighters
		n = 105
		Age: 29.4 ± 7.97 yrs
		Height: 1.77 ± 0.07 m
		Weight: 87.8 ± 15.6 kg
		BMI: 28.1 ± 4.4 kg/m^2^
Regehr et al., 2008 [44]	Canada	Police recruits
		n = 84
		Age: 30.3 ± 6.0 yrs
Sanchez-Molina et al., 2017 [45]	Spain	Soldiers of the Spanish Army
		n = 19
		Age: 31.9 ± 6.2 yrs
		Height: 173.6 ± 5.3 cm
		Weight: 73.8 ± 8.3 kg
		BMI: 24.4 ± 2.3 kg/m^2^
		Experience: 12.8 ± 7.0 yrs
Sanchez-Molina et al., 2019 [46]	Spain	Soldiers of the Spanish Army
		n = 24
		Age: 35.6 ± 6.6 yrs
		Height: 177.2 ± 7.4 cm
		Weight: 82.3 ± 11.0 kg
		BMI: 15.2 ± 7.4 kg/m^2^
Sanchez-Molina et al., 2018 [47]	Spain	Male soldiers of the Spanish Army
		n = 19
		Age: 30.2 ± 5.2 yrs
		Height: 176.1 ± 8.3 cm
		Weight: 77.9 ± 10.2 kg
		BMI: 25.0 ± 3.1 kg/m^2^
		Experience: 9.9 ± 5.2 yrs
Szivak et al., 2018 [8]	USA	Active-duty males in U.S Navy and Marine Corps
		n = 20
		Age range: 18–35 yrs
Takeyama et al., 2010 [48]	Japan	Firefighters
		n = 11
Taylor et al., 2007 [16]	USA	Male active-duty Navy personnel
		n = 19
		Age: 21.5 ± 1.7 yrs
		BMI: 24.2 ± 1.6 kg/m^2^
		Average time in service: 1.5 ± 0.9 yrs
Tornero-Aguilara et al., 2018 [49]	Spain	Professional soldiers of the Spanish Army
		n = 54
		Age: 30.6 ± 4.6 yrs
		Height: 175.8 ± 8.2 cm
		Weight: 82.0 ± 13.4 kg
		Civilians (used as control)
		n = 16
		Age: 26.0 ± 3.0 yrs
		Height: 174.2 ± 2.9 cm
		Weight: 77.0 ± 9.0 kg
Tornero-Aguilara et al., 2017 [50]	Spain	Soldiers of the Spanish Army
		n = 40
		Elite: Age: 28.5 ± 6.3 yrs
		Height: 178.4 ± 6.1 cm
		BMI: 25.1 ± 3.1 kg/m^2^
		Non-elite: Age: 31.9 ± 6.2 yrs
		Height: 173.6 ± 5.3 cm
		BMI: 24.5 ± 2.4 kg/m^2^

**Table 3:** Data extracted from included studies.

Study	Stressor	Duration of Event	Outcome Measures	Timepoints	Results		
					Pre	Post	*p*
Backe 2009 [20]	Mobile intensive care unit (MICU) and patient transport (PTA) in ambulance personnel	Two consecutive days; first day on-duty in MICU, following day working PTA	1. Salivary cortisol 2. HR	1. before, immediately after, and 30 min after tasks 2. continuously with Polar HR monitor	1. Morning rise cortisol was significantly higher going into MICU on day 1 (5.6 ± 40) than PTA on day 2 (3.5 ± 3.7), 2. HR during emergency and transport operations was significantly higher in MICU (30 ± 17) than PTA (7 ± 8)		1. *p* = 0.034 2. *p* = 0.000
Bustamante-Sanchez et al., 2020 [21]	Night and instrument helicopter flights	Night flights: 90.6 ± 13.3 min, instrument flights: 94.2 ± 8.9 min	1. Blood lactate (mmol/L), 2. Rating of Perceived Exertion (RPE), 3. Subjective Stress Perception (SSP), 4.Cortical Arousal (CFFT (Hz), 5. Blood Oxygen Saturation (%), 6. Body Temp (BT), 7. Urine pH, 8. Competitive State Anxiety Inventory (CSAI-2R), 9. State–Trait Anxiety Inventory (STAI)	All variables were recorded before and immediately following flights	1. 3.6 (mmol/L) * 2. 6.0 * 3. 20.0 * 4. 33.8 Hz * 5. 97.0% * 6. 36.7 (°C) * 7. 6.0 * 8. Instrument flight: 28.0, night flight: 20.5 9. Instrument flight: 11.5, night flight: 8.0 * values represent ALL flights	1. 7.2 (mmol/L) * 2. 9.0 * 3. 20.0 * 4. 33.2 Hz * 5. 97.0% * 6. 36.6 (°C) * 7. 6.0 * 8. Instrument flight: 23.0, night flight: 22.3 9. Instrument flight: 8.0, night flight: 8.5 * values represent ALL flights	1. *p* = 0.005 2. *p* = 0.030 3. *p* = 0.614 4. *p* = 0.584 5. *p* = 0.517 6. *p* = 0.445 7. *p* = 0.868 8. Instrument: *p* = 0.027, night: *p* = 0.475 9. Instrument: *p* = 0.859, night: *p* = 0.293
Charles et al., 2016 [22]	Shiftwork in police officers	Day shift, afternoon shift, and night shift	Salivary cortisol (measured as diurnal cortisol parameters across shiftwork) 1. AUCI = area under th curve with respect to increase against baseline 2. Slope of fitted regression line to the seven diurnal cortisol values 2. Slope of fitted regression line to four diurnal samples (excludes 15- 30- and 45-min samples)	Collected upon wakening, 15, 30, and 45 min after waking, at lunch, dinner, and bedtime	1. Day shift: −5316.20 ± 7205.2, Afternoon shift: −4911.18 ± 7414.4, Night shift: −4150.97 ± 6684.6 2. Day shift: −0.00328 ± 0.0017, Afternoon shift: −0.00321 ± 0.00017, Night shift: −0.00242 ± 0.0019 3. Day shift: −0.00278 ± 0.0018, Afternoon shift: −0.00245 ± 0.0019, Night shift: −0.00185 ± 0.0019		1. *p* = 0.518 *, *p* = 0.252 **, *p* = 0.475 ***, *p* = 0.663 *^ 2. *p* = 0.001 *, *p* = 0.001 **, *p* = 0.003 ***, *p* = 0.744 *^ 3. *p* = 0.002 *, *p* = 0.001 **, *p* = 0.029 ***, *p* = 0.159 *^ * *p* value comparing across three shift categores ** Day vs. Night *** Afternoon vs. Night *^ Day vs. Afternoon
Chester et al., 2013 [23]	Environmental Survival Training Course	15 days	1. Creatine Kinase (μ/L) 2. Profile of Mood States (POMS) 3. Kessler-10 4. Depression Anxiety Stress Scale 5. Serum IL-6 (pg/mL)	All variables assessed at baseline, day 5, day 11, and end of day 15	1. 148.7 ± 97.0 2. 87.4 ± 7.0 3. 12.0 ± 1.4 4. 0.9 ± 1.6 5. 94.3 ± 121.5	1. 339.5 ± 123.5 2. 99.5 ± 20.5 3. 16.7 ± 5.9 4. 8.2 ± 7.4 5. 156.4 ± 212.2	1. *p* < 0.01 2. NR 3. NR 4. NR 5. NR
Clemente-Suarez et al., 2016 [24]	Tactical combat parachute jump	NR	1. BOS (%) 2. HR (bpm) 3. Salivary cortisol (nmol/L) 4. Blood glucose (mmol/L) 5. Blood lactate (mmol/L) 6. Creatine Kinase (UI/L) 7. Cortical Arousal (Hz) 8. Competitive State Anxiety Inventory—State Anxiety (CSAI-2R)	All variables assessed in the morning for baseline and 15–20 min after landing	1. Novel: 97.4, Experienced: 97.1 2. Novel: 80.2 ± 13.6, Experienced: 69.9 ± 14.1 3. Novel: 5.8 ± 3.4, Experienced: 6.6 ± 3.2 4. Novel: 10.2 ± 1.1, Experienced: 10.1 ± 1.3 5. Novel: 1.1 ± 0.4, Experienced: 1.2 ± 0.3 6. Novel: 99.8 ± 63.4, Experienced: 118.3 ± 108.0 7. Novel: 39.3 ± 3.5, Experienced: 37.5 ± 4.1 8. Novel: 12.4 ± 6.2, Experienced: 5.2 ± 4.3	1. Novel: 96.2, Experienced: 96.4 2. Novel: 95.3 ± 12.7, Experienced: 81.9 ± 18.1 3. Novel: 8.8 ± 4.8, Experienced: 7.5 ± 3.8 4. Novel: 19.9 ± 1.2, Experienced: 9.8 ± 1.2 5. Novel: 5.5 ± 2.6, Experienced: 3.7 ± 1.6 6. Novel: 189.5 ± 125.6, Experienced: 162.5 ± 108.6 7. Novel: 39.8 ± 2.1, Experienced: 38.8 ± 4.1 8. Novel: 5.9 ± 5.0, Experienced: 1.8 ± 2.1	1. Novel: *p* = 0.064, Exp: *p* = 0.135 2. Novel: *p* = 0.001, Exp: *p* = 0.006 3. Novel: *p* = 0.047, Exp: *p* = 0.208 4. Novel: *p* = 0.324, Exp: *p* = 0.274 5. Novel: *p* < 0.001, Exp: *p* < 0.001 6. Novel: *p* = 0.348, Exp: *p* = 0.957 7. Novel: *p* = 0.484, Exp: *p* = 0.141 8. Novel: *p* = 0.004, Exp: *p* = 0.003
Clemente-Suarez et al., 2017 [25]	Melee combat situation	NR	1. HR (bpm) 2. Blood lactate (mmol/L) 3. HRV (SDSD in Ms) 4. Rating of Percevied Exertion (RPE) 5. Cortical Arousal (Hz)	All variables assessed 1 h before and immediately after exercise	1. 62.1 ± 14.7 2. 3.05 ± 1.05 3. 137.8 ± 89.1 4. 15.6 ± 2.5 5. 36.32 ± 4.01	1. 131.9 ± 10.7 2. 9.28 ± 2.20 3. 89.0 ± 45.7 4. NR 5. 35.97 ± 3.61	1. *p* = 0.000 2. *p* = 0.000 3. *p* = 0.114 4. NR 5. *p* = 0.564
Gomez-Oliva et al., 2019 [26]	Sanitary-military tasks while wearing nuclear, biological, and chemical equipment (NBC)	NR	1. Blood glucose 2. RPE 3. HR (bpm) 4. HRV (RMSSD) 5. Body temperature (°C)	Variables assessed right before and after accomplishing each task	1. With NBC: 100.8 ± 16.3, Without NBC:97.7 ± 9.9 2. With NBC: 6.3 ± 0.9, Without NBC: 6 3. With NBC:69.7 ± 13.4, Without NBC: 64.7 ± 10.7 4. With NBC: 46.3 ± 29.9, Without NBC: 57.6 ± 35.2 5. With NBC: 36.4 ± 0.4, Without NBC: 36.3 ± 0.3	1. With NBC: 100.7 ± 11.9, Without NBC: 87.4 ± 12.7 2. With NBC: 10.8 ± 3.4, Without NBC: 8.6 ± 1.8 3. With NBC: 72.8 ± 14.3, Without NBC: 63.5 ± 6.9 4. With NBC:46.2 ± 38.1, Without NBC: 49 ± 27 5. With NBC: 36.1 ± 0.6, Without NBC: 36.3 ± 0.4	1. With NBC: *p* = 0.093, Without NBC: *p* = 0.019 2. With NBC: *p* = 0.005, Without NBC: *p* = 0.007 3. With NBC: *p* = 0.170, Without NBC: *p* = 0.905 4. With NBC: *p* = 0.287, Without NBC: *p* = 0.059 5. With NBC: *p* = 0.107, Without NBC: *p* = 0.959
Hamarsland et al., 2018 [27]	Special Forces selection course	1 week	1. Serum testosterone (nmol/L) 2. Serum cortisol (nmol/L) 3. Serum SHBG (nmol/L) 4. Serum CK (μ/L) 5. Serum CRP (mg/L) 6. Serum TSH (mU/L) 7. Serum T3 (pmol/L) 8. Serum T4 (pmol/L) 9. Serum IGF-1 (nmol/L)	Testing conducted before and 0, 1, 3, 7, and 14 days after the course	Baseline 1. 13.4 ± 5.1 2. 493 ± 116 3. 27.3 ± 8.0 4. 633 ± 437 5. 1.6 ± 1.8 6. 1.5 ± 0.8 7.6.5 ± 0.3 8. 18.1 ± 2.4 9. 39.0 ± 8.7	0 h after cessation of course 1. 3.1 ± 1.2 2. 1122 ± 260 3. 40.1 ± 12.3 4. 2210 ± 1359 5. 23.1 ± 14.8 6. 1.9 ± 1.0 7. 4.1 ± 0.7 8. 14.5 ± 2.4 9. 14.6 ± 3.6	All outcome measures, except TSH, are significantly different following cessation of the selection course than at baseline. Actual *p* values not reported
Hormeno-Holgado et al., 2018 [28]	Last phase of a 10-week special operations course	4 days	1. Subjective Stress Perception 2. Fatigue Subjective Perception 3. RPE 4. Cortical arousal (Hz) 5. Body temperature (°C) 6. BOS (%) 7. HR (bpm) 8. Urine pH 9. Perceived Stress Scale 10. State-Trait Anxiety Inventory—State Anxiety	All variables assessed before and immediately after the 4 days	1. 67.4 ± 15.3 2. 64.4 ± 16.3 3. 15.0 ± 9.1 4. 33.9 ± 2.8 5. 36.5 ± 1.1 6. 98.0 ± 1.1 7. 80.7 ± 16.4 8. 5.9 ± 0.6 9. 20.3 ± 7.2 10. 21.7 ± 10.1	1. 77.9 ± 11.3 2. 80.1 ± 9.5 3. 16.1 ± 2.0 4. 31.5 ± 4.3 5. 36.5 ± 0.5 6. 98.7 ± 0.7 7. 82.9 ± 12.7 8. 5.4 ± 0.5 9. 21.2 ± 7.4 10. 10.6 ± 6.6	1. *p* = 0.001 2. *p* = 0.001 3. *p* = 0.442 4. *p* = 0.000 5. *p* = 0.870 6. *p* = 0.001 7. *p* = 0.503 8. *p* = 0.001 9. *p* = 0.253 10. *p* = 0.001
Horn et al., 2019 [29]	Three different training fire environents (Pallet, oriented strand board, and simulated fire/smoke)	NR	1. Core body temperature (°C) 2. HR (bpm)	1. Continuous measure with ingested core temp capsule 2. Continuous measure with HR monitoring bioharness	1. Mean core temp for firefighters was 38.46 (Pallet), 38.74 (OSB) and 38.47 (Fog). Mean core temp for intructors was 38.53 (Pallet), 38.46 (OSB), and 38.31 (Fog). 2. Mean HR for firefighters was 180.3 (Pallet), 181.2 (OSB), and 176.9 (Fog). Mean HR for instructors was 166.7 (Pallet). 169.4 (OSB), and 153.4 (Fog).	NR	NR
Hunt et al., 2019 [30]	Firefighting scenario-based activities while wearing turnout gear and breathing apparatus	63 ± 11 min	1. Core body temperature (°C) 2. HR (bpm) 3. Skin temperature (degreees C) 4. Physiological Strain Index (PSI) 5. Adaptive Physiological Strain Index (aPSI)	1. Continuous measure with ingested core temp capsule 2. Continuous measure with HR monitoring bioharness 3. Measured before and after 4–5. PSI and aPSI were calculated after all activities were complete	1. 37.4 ± 0.2 2. 89.9 ± 12.4 3. 32.9 ± 2.0 4. 2.7 ± 0.4 5. 2.8 ± 0.6	* values recorded at peak during work 1. NR 2. NR 3. NR 4. 7.3 ± 1.6 5. 8.2 ± 2.0	1. NR 2. NR 3. NR 4. *p* < 0.001 5. *p* < 0.001
Iizuka et al., 2012 [31]	Pilots in flight during a stressful working environment	NR	1. State anxiety (STAI-S) 2. Trait anxiety (STAI-T) 3. Salivary alpha-amylase	All variables assessed at baseline, 90 min pre flight and 30 min post flight	1. 41.3 ± 5.9 2. 39.2 ± 5.0 3. 31.1 ± 31.1	1. Pre-flight: 49.7 ± 3.2, post-flight: 41.6 ± 6.1 2. NR 3. Pre-flight: 49.0 ± 39.8, post-flight: 52.2 ± 42.7	1. Pre-flight: *p* < 0.05 2. NR 3. NR
Izawa et al., 2016 [32]	3-day rotating shift work with 24 h work shifts	24 h shift, followed by two off days	1. Salivary cortisol (log nmol/L) 2. Salivary CRP (log pmol/L) 3. Effort–Reward Imbalance (ERIQ)	Saliva samplings conducted twice a day, morning and evening	24 h work shift 1. Day 1 (0900): 1.30 ± 0.02, Day 1 (1900) 0.85 ± 0.03, Day 2 (0900): 1.24 ± 0.02 2. Day 1 (0900): NR, Day 1 (1900): 2.10 ± 0.03, Day 2 (0900): 2.09 ± 0.03	Work-free day 1. Day 2 (1900): 0.81 ± 0.03, Day 3 (0900): 1.19 ± 0.02, Day 3 (1900): 0.83 ± 0.02 2. Values for CRP not reported on 2nd day	1–2. Significant difference detected in morning cortisol levels across 2 days (*p* = 0.014) with levels on the 3rd morning lower than previous two 3. Higher effort scores (*p* = 0.031)and effort–reward ratios (*p* = 0.080) associated with lower cortisol levels
Kaikkonen et al., 2017 [33]	24 h work shift with a 6 h period of ambulance and emergency services and 6 h period of rescue services	24 h work shift	1. HR max 2. Stressindex 3. Recovery % 4. RMSSD (ms)	HR data recorded using thoracic belt during shift	1. 24 h shift: 156 ± 16, 6 h rescue: 136 ± 25, 6-h ambulance: 120 ± 14 2. 24 h shift: 108 ± 33, 6 h rescue: 118 ± 40, 6 h ambulance: 105 ± 36 3. 24 h shift: 27 ± 11, 6 h rescue: 12 ± 14, 6 h ambulance: 28 ± 25 4. 24 h shift: 42 ± 14, 6 h rescue: 38 ± 16, 6 h ambulance: 45 ± 21	NR	1. NR 2. Significantly higher during rescue service period than others (*p* < 0.01) 3. Significantly lower during rescue service period than others (*p* < 0.01) 4. Significantly lower during rescue service period than others (*p* < 0.01)
Kesler et al., 2017 [34]	7 trials involving different combos of SCBA size and design during various firefighting activities and work cycles	1 bout (19 min total), 2 bouts with rest (38 min total), 2 bouts back-to-back no rest (35 min total) in the standard 60 min cylinder	1. Peak HR (bpm) 2. Peak Core temperature (°C) 3. Perceptual stress—breathing (7 point scale) 4. Perceptual stress—feelings (11 point scale) 5. Perceptual stress—thermal sensations (8 point scale) 6. Perceptual stress—perceived exertion (15 point, 6–20 Borg scale)	1–2. Measured throughout with metabolic monitoring equipment 3–6. Measured before and after tasks/bouts	1. One bout: 182.0 ± 2.2, Two bouts: 186.8 ± 2.3, Back-to-back: 189 ± 2.3 2. One bout: 38.5 ± 0.08, Two bouts: 38.88 ± 0.14, Back-to-back: 39.03 ± 0.12 3. One bout: 1.23 ± 0.09, Two bouts: 1.10 ± 0.06, Back-to-back: 1.17 ± 0.7 4. One bout: 3.58 ± 0.20, Two bouts: 3.65 ± 0.18, Back-to-back: 3.60 ± 0.23 5. One bout: 4.27 ± 0.09, Two bouts: 4.05 ± 0.10, Back-to-back: 4.10 ± 0.13 6. NR—post-recording only	1. NR—continuous recording 2. NR—continuous recording 3. One bout: 3.77 ± 0.13, Two bouts: 4.47 ± 0.13, Back-to-back: 4.47 ± 0.16 4. One bout: 0.97 ± 0.33, Two bouts: −1.23 ± 0.44, Back-to-back: −1.60 ± 0.40 5. One bout: 6.00 ± 0.10, Two bouts: 6.69 ± 0.10, Back-to-back: 6.85 ± 0.11 6. One bout: 15.8, Two bouts: 18.0 Back-to-back: 18.1	1–6. *p* < 0.05
Ledford et al., 2020 [35]	First phase of Navy SEAL training	First two months of SEAL training, referred to as “First Phase”	1. Connor–Davidson Resilience Scale 2. Serum cortisol (μg/dL) 3. Serum DHEA (ng/mL) 4. DHEA/Cortisol ratio 5. Serum BDNF (pg/mL)	Each outcome measure assessed at the beginning of SEAL training to determine dropout	1. Enrolled: 83.66, Dropped: 82.64 2. Enrolled: 13.15, Dropped: 11.96 3. Enrolled: 2.20, Dropped: 1.93 4. Enrolled: 0.21, Dropped: 0.16 5. Enrolled: 230.98, Dropped: 225.30	NR	1. *p* = 0.51 2. *p* = 0.28 3. *p* = 0.00 4. *p* = 0.00 5. *p* = 0.73
Lieberman et al., 2016 [15]	Simulated captivity in military survival training (SERE)	3 separate SERE school classes over a three-month period	1. Serum cortisol (ug/dL) 2. Serum epinephrine (pg/mL) 3. Serum norepinephrine (pg/mL) 4. Serum DHEA-sulfate (ug/dL) 5. Serum testosterone (ng/dL) 6. Serum sTfR 7. Salivary cortisol (ug/mL) 8. Salivary NPY (pmol/L) 9. Salivary DHEA-sulfate (pg/mL) 10. Salivary testosterone (pg/mL) 11. HR (bpm) 12. Profile of Mood States	1–6. Collected at baseline (B), after mock interrogation 1 (M1) and mock interrogation 2 (M2), and at recovery (R) 7–10. Collected at baseline, after 2 interrogations, and at recovery 11. Collected every ~2 h during baseline, interrogation 1 and interrogation 2 12. Assessed at baseline, after each interrogation (2), and at recovery	1. B: 20.103 ± 0.38 ^~#, M1: 23.095 ± 0.75 *~# 2. B: 41 ^~#, M1: 82 *# 3. B: 403 ^~#, M1: 905 * 4. B: 290.431 ± 12.66 ^~#, M1: 519.19 ± 23.39 *~ 5. B: 472 ^~#, M1: 84 *~# 6. B:18.966 ± 0.52 ^~#, M1: 19.724 ± 0.54 7. B: 0.043, M1: 0.249 8. B: 79.70, M1: 86.20 9. B: 1595, M1: 3049 10. B: 42.7, M1: 34.8 11. B: 68.7, M1: 97.6 12. Exact values NR (2, 3, 5, and 6–9 are Median values)	1. M2: 27.234 ± 0.82 *^#, R: 18.033 ± 0.59 *^~ 2. M2: 87 *#, R: 66 *^~ 3. M2: 944 *, R: 785 *~ 4. M2: 562.121 ± 26.07 *^#, R: 542.914 ± 23.8 *~ 5. M2: 177 *^#, R: 157 *^~ 6. M2: 20.121 ± 0.53 *#, R: 19.241 ± 0.49 ~ 7. M2: 0.270, R: 0.133 8. M2: 117.55, R: 106.50 9. M2: 3932, R: 3427 10. M2: 46.5, R: 43.1 11. M2: 124.6 12. Exact values NR	1–6. * = Significantly different from baseline. ^ = Significantly different from Mock Interrogation 1. ~ = Significantly different from Mock Interrogation 2. # = Significantly different from End of SERE (recovery) 7–10: “All saliva biomarkers were significantly different over the collection periods (*p* < 0.001)” 11. *p* < 0.001 12. “All mood states changed significantly over the course of training (*p* < 0.001)”
Marcel-Millet et al., 2018 [36]	Simulated fire rescue intervention while wearing PPC, SCBA, and SCBAc	≥1 h	1. Mean HR (%HR max) 2. Mean Breathing Frequency (breaths/min) 3. RPE 4. HRV—SDNN 5. HRV—RMSSD	1–3. Parameters were continuously recorded during the intervention using a connected suit 4–5. Recorded during 5 and 10 min rest and recocery periods	(during) 1. PPC: 79.5 ± 5.3, SCBAc: 83.2 ± 4.1, SCBA: 83.1 ± 5.2 2. PPC: 40.4 ± 5.6, SCBAc: 43.5 ± 5.6, SCBA: 34.1 ± 5.9 3. PPC: 5.9 ± 1.5, SCBAc: 6.9 ± 1.2, SCBA: 7.8 ± 1.3 4. NR 5. NR	(after) 1. NR 2. NR 3. NR 4. PPC: 27.8 ± 14.1, SCBAc: 21.7 ± 13.1, SCBA: 21.4 ± 9.2 5. PPC: 2.1 ± 0.5, SCBAc: 2.0 ± 2.5, SCBA: 2.0 ± 0.5	1. SCBAc and SCBA values significantly greater than PPC (*p* < 0.0001) 2. SCBA value is significantly less than PPC (*p* < 0.0001) 3. SCBA and SCBAc values significantly greater than PPC (*p* < 0.05) 4. SCBA value significantly less than PPC (*p* < 0.05) 5. NR
Marins et al., 2018 [37]	Occupational Physical Ability Test (OPAT) while carrying load (PPE)	1 h	1. HRV—LF 2. HRV—HF 3. HRV—RMSSD 4. RPE 5. Blood lactate concentration (nmol/L)	1–3. HR variables collected continiously with cardiac monitor 4. Collected post-OPAT 5. Collected before, immediately after, and 3 and 5 min after OPAT	1. No PPE: 596.6, With PPE: 964.2 2. No PPE: 235.2, With PPE: 389 3. No PPE: 32.0, With PPE: 28.8 4. NR 5. No PPE: 1.7, With PPE: 1.7	1. 2. 3. 4. No PPE: 8.6 ± 0.8, With PPE: 8.7 ± 1.0 5. No PPE: 11.7 + 2.7, With PPE: 9.6 ± 1.5	1. *p* = 1.0 2. *p* = 0.6 3. *p* = 0.9 4. *p* = 0.77 5. *p* < 0.05 (between conditions)
McClung et al., 2013 [38]	7-day winter military training exercise culminating in 3-day 54 km ski march	7 days	1. Hemoglobin (d/dL) 2. Serum ferritin (ng/mL) 3. Serum sTfR (nmol/L) 4. Serum hepcidin (pg/mL) 5. Serum IL-6 (ng/mL)	Blood samples assessed as baseline, following a 4-day pre-march training period, and immediately after completion of a 3-day ski march	1. 14.7 ± 0.8 2. 109.2 ± 44.1 3. 18.9 ± 4.2 4. 6.5 ± 3.5 5. 9.1 ± 4.9	1. 14.1 ± 0.9 2. 133.0 ± 55.2 3. 18.7 ± 3.2 4. 10.2 ± 6.9 5. 14.5 ± 8.4	Significant differences exist between all Baseline and POST values (*p* < 0.05), with the exception of sTfR
Nindl et al., 2007 [39]	8-week (62 days) U.S Army Ranger Training Course	62 days	1. Serum cortisol (nmol/L) 2. Serum testosterone (nmol/L) 3. Serum IGF-1 (ng/mL)	All variables assessed the morning before (fasted) and immediately following the end of the course	1. 469 ± 106 2. 17.3 ± 4.8 3. 239 ± 80	1. 692 ± 109 2. 3.0 ± 1.8 3. 108 ± 29	1. *p* < 0.001 2. *p* < 0.001 3. *p* < 0.001
Ojanen et al., 2018 [40]	21-day military field training	21 days	1. Serum testosterone (nmol/L) 2. Serum cortisol (nmol/L) 3. Serum IGF-1 (pmol/L) 4. Serum SHBG (nmol/L)	Variables assessed for baseline values 1 week before MFT, on day 12, on the last day of training, and following a recovery period of 4 days	1. PRE: 18.4 ± 4.5, MID: 13.8 ± 4.9 ** 2. PRE: 301 ± 86, MID: 355 ± 76 * 3. PRE: 40.6 ± 7.7, MID: 32.5 ± 8.9 ** 4. PRE: 30.1 ± 7.6, MID: 32.8 ± 7.9 **	1. POST: 16.0 ± 4.2 **^^, RECO: 19.9 ± 3.7 *^^++ 2. POST: 396 ± 69 **^^, RECO: 385 ± 85 ** 3. POST: 32.5 ± 7.7 **, RECO: 39.4 ± 7.8 ^^++ 4. POST: 34.3 ± 9.1 **, RECO: 31.5 ± 8.1 ++	* = *p* < 0.05 **, ^^, ++ = *p* < 0.01 * = compared with PRE values ^ = compared wist MID values + = compared with POST values
Ojanen et al., 2018 [41]	Prolonged military field training	21 days	1. Serum IGF-1 (pmol/L) 2. Serum TNF-alpha (ng/mL) 3. Leptin (ng/mL) 4. Serum IL-6 (ng/mL) 5. Creatine Kinase (U/L)	Variables assessed for baseline values 1 week before MFT and again on day 13, day 22, and following a period of 4 days recovery	1. PRE: 40.5 ± 7.8, MID: 31.8 ± 8.4 *** 2. PRE: 9.4 ± 1.9, MID: 10.3 ± 3.7 ** 3. PRE: 3.8 ± 2.8, MID: 1.3 ± 1.1 *** 4. PRE: 1.8 ± 2.8, MID: 2.0 ± 4.9 5. PRE: 106 ± 95. MID: 198 ± 88 ***	1. POST: 32.6 ± 7.6 ***^^^, RECO: 38.9 ± 7.7 +++ 2. POST: 7.1 ± 1.7 ***^^, RECO: 8.5 ± 1.6 *^^ +++ 3. POST: 2.1 ± 1.6 ***^^^, RECO: 3.4 ± 3.0 ^^^+++ 4. POST: 1.4 ± 2.1, RECO: 1.2 ± 2.2 5. POST: 141 ± 63 *^^	* = *p* < 0.05 **, ^^ = *p* < 0.01 ***, ^^^, +++ = *p* < 0.001 * = compared with PRE values ^ = compared wist MID values + = compared with POST values
Perroni et al.,. 2009 [42]	Simulated rescue firefighting intervention while wearing standard turnout gear	704 ± 135 s	1. State and trait anxiety (STAI) 2. Profile of Mood States 3. Salivary alpha-amylase (U/mL) 4. Salivary cortisol (nmol/L)	1. Trait anxiety (Y2) was assessed on a rest day before. State anxiety (Y1) was assessed immediately before and after intervention 2. Immediately before and after intervetion 3–4. Saliva samples were collected in the morning for baseline levels, immediately before intervention, and 30 and 90 min after	1. Baseline Y1: 30.5 ± 5.3, Y2: 30.8 ± 4.9 2. Tension/anxiety: 6 ± 1, Confusion/bewilderment: 5 ± 1, Fatigue/intertia: 3 ± 1, Anger/hostility: 3 ± 1, Depression/dejection: 2 ± 1, Vigour/activity: 17 ± 1 3. Rest day morning: 64.2 ± 10.9, Rest day afternoon: 133.9 ± 30.3, Before intervention: 102.3 ± 18.7 4. Rest day morning: 16.7 ± 2.2, Rest day afternoon: 2.7 ± 0.4, Before intervetion: 11.3 ± 1.9	1. NR 2. NR 3. 30 min after: 279.7 ± 59.0 4. 30 min after: 22 ± 3	1. “no significant change in anxiety scores were observed” 2. “no differences emerged between pre- and post-intervention in POMS subscales” 3. *p* = 0.0012 4. *p* < 0.001
Petruzzello et al., 2014 [43]	Short-term live firefighting intervention wearing PPE and SCBA	18 min, with nine two-minute periods of alternating rest/work cycles	1. Core temperature (°C) 2. HR (bpm) 3. Perception of thermal sensations (TS) 4. Perception of respiratory distress (RD) 5. RPE 6. Feelings scale (very bad to very good) 7. State/Trait Anxiety Inventory—State	1. Continuous monitoring with ingested capsule 2. Continious monitoring through Polar HR monitor 3–7. All subjective measures completed just before and within 20–30 min after the intervention	1. Career: 37.59 ± 0.31, Volunteer: 37.59 ± 0.43 2. Career: 86.40 ± 17.28, Volunteer: 101.68 ± 17.66 3. Career: 4.2 ± 0.7, Volunteer: 4.5 ± 0.6 4. Career: 1.1 ± 0.3, Volunteer: 1.2 ± 0.5 5. NR 6. Career: 4.1 ± 1.1, Volunteer: 3.5 ± 1.3 7. Career: 14.88 ± 3.2, Volunteer: 16.1 ± 3.9	1. Career: 38.22 ± 0.36, Volunteer: 38.30 ± 0.94 2. Career: 165.38 ±, Volunteer: 173.17 ± 16.84 3. Career: 5.7 ± 0.7, Volunteer: 6.0 ± 1.0 4. Career: 2.4 ± 1.1. Volunteer: 3.1 ± 1.1 5. Career: 14.2, Volunteer: 14.9 6. Career: 2.7 ± 1.6, Volunteer: 1.1 ± 1.9 7. Career: 15.9 ± 3.7, Volunteer: 17.1 ± 3.4	Values reported as Effect Size 1. Career: 1.89, Volunteer: 0.98 2. Career: 5.41, Volunteer: 4.18 3. Career: 2.16, Volunteer: 1.84 4. Career: 1.63, Volunteer: 2.25 5. NR 6. Career: −1.03, Volunteer: −1.49 7. Career: 0.28, Volunteer: 0.28
Regehr et al., 2008 [44]	High-fidelity simulation of a policing event	NR	1. HR (bpm) 2. Salivary cortisol (nmol/L) 3. State/Trait Anxiety Inventory (STAI) 4. Self-evaluation of performance	1. Continuosly measured throughout event 2. Baseline assessed prior to event. Then obtained again 20 and 30 min after event 3. Obtained immediately following event and again 20 min after 4. Performed following the event	NR; pre- and post-simulation measures were not examined, only assoications/correlations between measures	NR	1. Cortisol level 20 min after event was significantly correlated with both relative ranking of performance (*p* = 0.05) and the performance checklist (*p* = 0.04) 2. Significant differences exist between groups in cortisol levels at baseline (*p* < 0.001) and in peak levels following exposure (*p* < 0.002) 3. A significant difference exists in subjective distress change as measured by differences in scores of STAI (*p* < 0.05)
Sanchez-Molina et al., 2017 [45]	Combat parachute jump while equipped with full gear (appx 14 kg in total) and urban combat simulation	NR	1. RPE 2. Blood lactate (nmol/L) 3. Cortical activation (Hz) 4. HR (ppm) 5. HRV—RMSSD 6. HRV—LF 7. HRV—HF 8. BOS (%) 9. Skin Temperature (°C) 10. Competitive State Anxiety Inventory—Cognitive Anxiety 11. State/Trait Anxiety Inventory—State Anxiety	All variables were assessed before and immediately after the simulation	1. 6.00 ± 0.00 2. 1.27 ± 0.20 3. 37.68 ± 2.85 4. 64.18 ± 8.70 5. 47.70 ± 17.02 6. 63.45 ± 14.68 7. 36.55 ± 14.68 8. 97.45 ± 1.12 9. 36.88 ± 1.41 10. 4.8 ± 4.7 11. 5.00 ± 3.49	1. 10.22 ± 0.58 2. 7.01 ± 1.51 3. 37.95 ± 2.75 4. 94.36 ± 13.41 5. 30.74 ± 18.11 6. 77.24 ± 10.83 7. 22.76 ± 10.83 8. 97.18 ± 4.99 9. 36.26 ± 2.21 10. 3.3 ± 4.1 11. 9.90 ± 10.57	1. *p* = 0.003 2. *p* = 0.003 3. *p* = 0.624 4. *p* = 0.003 5. *p* = 0.003 6. *p* = 0.003 7. *p* = 0.003 8. *p* = 0.048 9. *p* = 0.475 10. *p* = 0.023 11. *p* = 0.139
Sanchez-Molina et al., 2019 [46]	Checkpoint simulation including surveillance, unexpected attacks, and melee combat	NR	1. RPE 2. HR (bpm) 3. BOS (%) 4. Blood glucose (mmol/L) 5. Blood lactate (mmol/L) 6. Skin temperature (°C) 7. Cortical arousal (Hz) 8. Competitive State Anxiety Inventory—Somatic Anxiety 9. State/Trait Anxiety Inventory—State Anxiety	All variables were assessed before and immediately after the simulation	1. 6.00 ± 0.00 2. 77.42 ± 12.73 3. 97.13 ± 0.61 4. 6.08 ± 0.76 5. 2.20 ± 0.43 6. 38.45 ± 0.52 7. 36.22 ± 0.33 8. 3.30 ± 2.73 9. 7.48 ± 6.84	1. 10.75 ± 1.11 2. 93.92 ± 16.63 3. 96.04 ± 1.00 4. 6.16 ± 0.99 5. 4.58 ± 2.91 6. 36.51 ± 1.16 7. 35.60 ± 0.33 8. 6.04 ± 4.55 9. 9.70 ± 6.90	1. *p* = 0.000 2. *p* = 0.000 3. *p* = 0.000 4. *p* = 1.00 5. *p* = 0.000 6. *p* = 0.000 7. *p* = 0.131 8. *p* = 0.000 9. *p* = 0.120
Sanchez-Molina et al., 2018 [47]	Urban combat simulation	NR	1. HR (bpm) 2. HRV—RMSSD 3. HRV—LF 4. HRV—HF 5. BOS (%) 6. Blood glucose (mmol/L) 7. Blood lactate (mmol/L) 8. Cortical activation (Hz) 9. Competitive State Anxiety Inventory—Somatic Anixety 10. State/Trait Anxiety Inventory—State Anxiety	All variables were assessed before and immediately after the simulation	1. HIU: 70.91 ± 13.71, LIU: 65.10 ± 9.75 2. HIU: 164.89 ± 73.16, LIU: 38.26 ± 40.69 ** 3. HIU: 19.25 ± 13.11, LIU: 76.60 ± 10.75 ** 4. HIU: 80.59 ± 13.41, LIU: 35.10 ± 25.51 ** 5. HIU: 97.25 ± 0.62, LIU: 95.89 ± 2.66 6. HIU: 98.00 ± 22.71, LIU: 94.63 ± 6.36 7. HIU: 1.39 ± 0.36, LIU: 1.20 ± 0.31 8. HIU: 40.28 ± 4.40, LIU: 36.55 ± 5.20 ** 9. HIU: 8.41 ± 5.08, LIU: 3.52 ± 3.68 10. HIU: 9.83 ± 5.95, LIU: 8.94 ± 9.34 HIU—Heavy Infantry Unit, LIU = Light Infantry Unit	1. HIU: 93.33 ± 9.86, LIU: 82.21 ± 10.20 ** 2. HIU: 29.32 ± 9.33, LIU: 7.62 ± 5.62 ** 3. HIU: 88.68 ± 4.45, LIU: 90.93 ± 3.42 4. HIU: 11.32 ± 4.45, LIU: 14.72 ± 13.36 5. HIU: 97.00 ± 1.04, LIU: 95.26 ± 1.62 ** 6. HIU: 101.30 ± 15.05, LIU: 97.47 ± 4.07 7. HIU: 4.99 ± 2.79, LIU: 4.45 ± 3.65 8. HIU: 40.08 ± 2.96, LIU: 35.31 ± 5.01 ** 9. HIU: 7.41 ± 3.17, LIU: 4.63 ± 3.84 ** 10. HIU: 11.41 ± 4.79, LIU: 9.00 ± 8.98	1.HIU: *p* = 0.000, LIU: *p* = 0.000 2. HIU: *p* = 0.002, LIU: *p* = 0.000 3. HIU: *p* = 0.002, LIU: *p* = 0.000 4. HIU: *p* = 0.002, LIU: *p* = 0.000 5. HIU: *p* = 0.527, LIU: *p* = 0.431 6. HIU: *p* = 0.610, LIU: *p* = 0.628 7. HIU: *p* = 0.002, LIU: *p* = 0.000 8. HIU: *p* = 0.873, LIU: *p* = 0.809 9. HIU: *p* = 0.046, LIU: *p* = 0.346 10. HIU: *p* = 0.282, LIU: *p* = 0.775 ** = significantly different than HIU values (*p* < 0.05)
Szivak et al., 2018 [8]	U.S Navy Survival, Evasion, Resistance, and Escape (SERE) training	10 days	1. Serum cortisol (nmol/L) 2. Serum testosterone (nmol/L) 3. Serum neuropeptide-Y (pg/mL) 4. Plasma epinephrine (pmol/L) 5. Plasma norepinphrine (pmol/L) 6. Plasma dopamine (pmol/L)	Blood samples were obtained at three timepoints: baseline (T1) (first day of SERE), a stress assessment (T2) (which ocurred 10 days after baseline) and a recovery assessment (T3) (which occurred 24 h after end of SERE)	1. T1: 122.70 ± 49.79; T2: 766.86 ± 157.87 * 2. T1: 14.83 ± 4.66; T2: 5.50 ± 4.06 * 3. T1: 348.16 ± 88.70; T2: 328.42 ± 139.56 4. T1: 348.74 ± 140.02; T2: 593.77 ± 205.39 * 5. T1: 2323.65 ± 458.67; T2: 6758.45 ± 2351.21 * 6. T1: 96.49 ± 30.78; T2: 275.72 ± 135.09 *	1. T3: 333.84 ± 128.30 *^ 2. T3: 6.81 ± 2.66 * 3. T3: 146.16 ± 47.47 *^ 4. T3: 343.57 ± 78.63 ^ 5. T3: 4218.90 ± 1420.80 *^ 6. T3: 172.71 ± 97.74 *^	* = Significant difference from baseline timepoint (T1) (*p* ≤ 0.05) ^ = Significant difference from stress timepoint (T2) (*p* ≤ 0.05)
Takeyama et al., 2010 [48]	17-day field study examining effects of shift schedules; 24 h every other day	17-day shfit work	1. HR (bpm) 2. HRV—HF 3. HRV—LF 4. HRV—LF/HF ratio 5. Cortical activation (Hz) 6. Oral temperatures 7. Fatigue questionnaire	1–4. Measured continuously with HR monitor 5–7. Each measure taken at ~2 h intervals during working days	1. HR during period 5 (P5) was significantly higher than those in other periods 2. LF/HF ratio was significantly lower in P4 than P5 3. No significant difference exist in oral temperature between periods 4. Fatigue complaints were significantly higher after night shift in P4 and at 0730 during shift periods 5. Physical projection complaints at 0730 during P4 were significantly higher than in P1, P2, and P5		1. *p* < 0.05 2. *p* < 0.05 3. NR 4. *p* < 0.05 5. *p* < 0.05
Taylor et al., 2007 [16]	12-day SERE survival course	12 days	1. Salivary cortisol (nmol/L) 2. Salivary DHEA (ng/mL) 3. DHEA-cortisol ratio 4. Dissociative Stress Scale (CADSS) 5. Impact of Events Scale (IES)	1–3. Salivary baseline measures were obtained over 2 consecutive days (morning and evening) prior to start of training (free-living), and again during the stressful captivity phase 4–5. Administered immediately after training	Values during free-living conditions 1. 9.2 ± 3.4 (at 0900) and 3.5 ± 3.0 (at 1930) 2. 1.7 ± 1.3 (at 0900) and 1.5 ± 0.8 (at 1930) 3. 0.2 ± 0.2 (at 0900) and 0.7 ± 0.09 (at 1930) 4. Exact values NR 5. Exact values NR	Values during stressful captivity 1. 18.4 ± 10.5 (at 0900) and 27.7 ± 10.9 (at 1930) 2. 6.7 ± 3.5 (at 0900) and 4.5 ± 3.0 (at 1930) 3. 0.4 ± 0.2 (at 0900) and 0.18 ± 0.2 (at 1930) 4. 28.5 ± 10.7 5. Exact values NR	1. *p* < 0.001 2. *p* < 0.001 3. Increased significantly from FL to SC at 0900, decresed significantly from FL to SC at 1930 (*p* < 0.01) 4. “dissociative symptoms did not relate to any endocrine changes during stressful captivity” 5. IES–Avoid and IES–Intrusion were positively associated with cortisol concentrations at 0900 (*p* < 0.05)
Tornero-Aguilera et al., 2018 [49]	Simulated underground operations, carrying all equipment (totaling 23.6 kg) SFV = Soldiers Fire Night Vision SNFNV = Soldiers No-fire No-Night Vision	NR	1. RPE 2. HR (bpm) 3. HRV—RMSSD 4. HRV—LF 5. HRV—HF 6. Blood lactate (mmol/L) 7. Cortical arousal (Hz) 8. Body temperature (°C) 9. BOS (%) 10. Competitive State Anxiety Inventory—Cognitive Anxiety 11. State/Trait Anxiety Inventory—State Anxiety	1. Assessed before and immediately following simulation 2–5. Continiously monitored through simulation with wearable 6–11. Assessed before and immediately following simulation	1. SFV: 6 ± 0, SNFNV: 6 ± 0 2. SFV: 78.6 ± 12.3, SNFNV: 63 ± 9.8 * 3. SFV: 218.54 ± 213.2, SNFNV: 341 ± 349.8 * 4. SFV: 77.78 ± 20.6, SNFNV: 76.5 ± 25.0 5. SFV: 22.0 ± 20.6, SNFNV: 39.1 ± 33.2 6. SFV: 3.98 ± 2.9, SNFNV: 3.5 ± 2.4 7. SFV: 37.4 ± 2.7, SNFNV: 37.09 8. SFV: 36.2 ± 0.9, SNFNV: 35.9 ± 1.3 * 9. SFV: 97.5 ± 0.8, SNFNV: 98.3 ± 0.8 * 10. SFV: 8.5 ± 3.6, SNFNV: 3.7 ± 2.5 * 11. SFV: 10.37 ± 8.5, SNFNV: 5.2 ± 3.2 * * significantly different from SFV value	1. SFV: 12.7 ± 2.4, SNFNV: 12.1 ± 2.6 2. SFV: 119.6 ± 13.8, SNFNV: 112 ± 21.1 3. SFV: 347.9 ± 232.2, SNFNV: 300.8 ± 256.8 4. SFV: 94.2 ± 3.4, SNFNV: 70.1 ± 27.8 ** 5. SFV: 11.09 ± 14.9, SNFNV: 31.2 ± 26.4 ** 6. SFV: 16.06 ± 4.9, SNFNV: 9.2 ± 3.5 ** 7. SFV: 36.1 ± 2.9, SNFNV: 36.04 8. SFV:34.3 ± 1.2. SNFNV: 36.3 ± 1.8 ** 9. SFV: 96.5 ± 0.7, SNFNV: 97 ± 0.09 10. SFV: 9.6 ± 1.8, SNFNV: 5.2 ± 3.2 11. SFV: 15.12 ± 9.7, SNFNV: 6.8 ± 4.2 ** ** significantly different from SFV value	1. SFV: *p* = 0.000, SNFNV: *p* = 0.005 2. SFV: *p* = 0.000, SNFNV: *p* = 0.000 3. SFV: *p* = 0.67, SNFNV: *p* = 0.643 4. SFV: *p* = 0.000, SNFNV: *p* = 0.769 5. SFV: *p* = 0.136, SNFNV: *p* = 0.985 6. SFV: *p* = 0.000, SNFNV: *p* = 0.000 7. SFV: *p* = 0.034, SNFNV: *p* = 0.278 8. SFV: *p* = 0.000, SNFNV: *p* = 0.159 9. SFV: *p* = 0.010, SNFNV: *p* = 0.000 10. SFV: *p* = 0.010, SNFNV: *p* = 0.000 11.SFV: *p* = 0.158, SNFNV: *p* = 0.001
Tornero-Aguilera et al., 2017 [50]	Tactical combat simulation	NR	1. Blood lactate (mmol/L) 2. Skin temperature (°C) 3. BOS (%) 4. HR (bpm) 5. Cortical arousal (Hz)	All variables were assessed before and immediately after the simulation	1. Elite: 1.4 ± 0.3, Non-elite: 1.2 ± 0.3 2. Elite: 36.7 ± 1.2, Non-elite: 34.2 ± 0.8 * 3. Elite: 97.4 ± 1, Non-elite: 97.5 ± 1.1 4. Elite: 64.4 ± 11.8, Non-elite: 82.9 ± 12.3 * 5. Elite: 36.1 ± 3.9, Non-elite: 37.1 ± 3.2 * values are significantly different than Elite	1. Elite: 6.6 ± 1.3, Non-elite: 3.8 ± 1.3 * 2. Elite: 36.4 ± 1.7, Non-elite: 37.1 ± 1.5 3. Elite: 96.4 + 3.8, Non-elite: 96.3 ± 1.4 4. Elite: 88 ± 13.8, Non-elite: 93.9 ± 12.8 5. Elite: 36.1 ± 3.15, Non-elite: 36.5 ± 8.9 * values are significantly different than Elite	1. Elite: *p* = 0.000, Non-elite: *p* = 0.000 2. Elite: *p* = 0.501, Non-elite: *p* = 0.356 3. Elite: *p* = 0.343, *p* = 0.003 4. Elite: *p* = 0.000, *p* = 0.002 5. Elite: *p* = 0.765, Non-elite: *p* = 0.734
Tornero-Aguilera et al., 2018 [49]	Tactical combat simulation	NR	1. HR (bpm) 2. HRV—RMSSD 3. HRV—HF 4. HRV—LF 5. RPE 6. Blood lactate (mmom/L) 7. Cortical arousal (Hz) 8. BOS (%) 9. Competitive State Anxiety Inventory—Cognitive Anxiety 10. State/Trait Anxiety Inventory—State Anxiety	1–4. Measured continuously with HR monitor 5–11. Assessed before and immediately after the simulation LTG = lower-trained group, HTG = higher-trained group	1. LTG: 69.0 ± 12.3, HTG: 65.11 ± 11.3 2. LTG: 34.4 ± 43.0: HTG: 33.9 ± 18.1 3. LTG: 78.9 ± 14.1, HTG: 81.5 ± 16.8 4. LTG: 21.8 ± 14.1, HTG: 18.9 ± 9.7 * 5. LTG: 6 ± 0, HTG: 6 ± 0 6. LTG: 1.3 ± 0.31, HTG: 1.4 ± 0.3 7. LTG: 38.0 + 2.9, HTG: 34.6 ± 4.1 * 8. LTG: 99.1 ± 1.0, HTG: 97.3 ± 1 * 9. LTG: 6.0 ± 3.7, HTG: 8.3 ± 3.2 10. LTG: 4.2 ± 4.0, HTG: 9.3 ± 8.7 * * values are significantly different than LTG	1. LTG: 96.5 ± 19.6, HTG: 89.8 ± 14 * 2. LTG: 46.9 ± 47.9, HTG: 39.5 ± 29.7 3. LTG: 79.1 ± 18.6, HTG: 75.6 ± 10.2 4. LTG: 27.2 ± 26.8, HTG: 24.4 ± 9.8 5. LTG: 11.7 ± 3.2, HTG: 9.9 ± 1.1 * 6. LTG: 2.5 ± 1.89, HTG: 6.8 ± 1.5 * 7. LTG: 37.1 ± 3.0, HTG: 34.5 ± 4.2 * 8. LTG: 96.9 ± 0.91, HTG: 96.4 ± 3.6 9. LTG: 5.8 ± 4.2, HTG: 6.7 ± 3.5 10. LTG: 3.9 ± 3.0, HTG: 15.5 ± 9.7 * * values are significantly different than LTG	1. LTG: *p* = 0.000, HTG: *p* = 0.000 2. LTG: *p* = 0.001, HTG: *p* = 0.003 3. LTG: *p* = 0.530, HTG: *p* = 0.000 4. LTG: *p* = 0.743, HTG: *p* = 0.000 5. LTG: *p* = 0.000, HTG: *p* = 0.000 6. LTG: *p* = 0.005, HTG: *p* = 0.000 7. LTG: *p* = 0.115, *p* = 0.952 8. LTG: *p* = 0.000, HTG: *p* = 0.265 9. LTG: *p* = 0.819, HTG: *p* = 0.043 10. LTG: *p* = 0.731, HTG: *p* = 0.036
Tyyska et al., 2009 [51]	15-day military field training	15 days	1. Serum testosterone (nmol/L) 2. Serum cortisol (nmol/L) 3. Serum SHBG (pmol/L)	Serum hormones were measured one day before training, as well as on day 8 and day 15 of training. All blood samples were taken in the morning	1. Day 1: 18.0 ± 3.0, Day 8: 17.2 ± 2.2 2. Day 1: 454 ± 90, Day 8: 485 ± 122 3. Exact values NR	1. Day 15: 18.8 ± 4.5 2. Day 15: 398 ± 101 3. Exact values NR	1. “no significant change” 2. “no significant change” 3. “no significant change in first 8 days, but significant increase from day 8 to day 15 (*p* = 0.015)”
Vaara et al., 2015 [52]	11-week paratrooper trainig period, including a 5-day military field training	11-weeks	1. Serum testosterone (nmol/L 2. Serum cortisol (pmol/L) 3. Serum IGF-1 (pmol/L) 4. Serum SHBG (pmol/L)	Serum hormones were measured at 2 weeks (M1), 4 weeks (M2), and 7 weeks (M3). Subjects then were involved in a 5-day strenuous MFT and hormones were assessed following training (week 9 -M4) and again 2 weeks after (M5)	1. M1: 13.0 ± 3.1, M3: 13.2 ± 4.5 2. M1: 514.7 ± 108.3, M3: 471.1 ± 95.4 * 3. M1: 32.3 ± 9.7, M3: 32.2 ± 7.8 4. M1: 34.8 ± 9.7, M3: 36.7 ± 9.7	1. M4: 7.0 ± 5.3 ^^, M5: 12.7 ± 4.7 2. M4: 491.2 ± 113.2 *, M5: 537.6 ± 131.5 3. M4: 23.1 ± 7.6 ^^, M5: 32.2 ± 8.8 4. M4: 43.4 ± 12.1 ^^, M5: 34.8 ± 10.4	* *p* < 0.05 compared with M5 only ^^ *p* < 0.05 compared separately with M1, M3, and M5
Vartanian et al., 2018 [53]	4-day military captivity survival training; Conduct After Capture (CAC)—Instructors	4 days x 2 consecutive courses (3 days rest between each course)	1. Multidimensional Fatigue Inventory 2. Profile of Mood States 3. Dissociative States Scale 4. Delayed Match-to-Sampling 5. Blood lactate (mmol/L) 6. Serum NPY (pg/mL) 7. Serum DHEA (ng/dL) 8. Serum IL-6 9. Serum Testosterone (nmol/L) 10. Serum cortisol (nmol/L)	All variables were collected on Day 1 (baseline) prior to start and Day 4 following completion of training. This was performed after the first and the second course.	The main effect of timepoint or week or the interaction between timepoint and week did not reached statistical significance for IL-6, Lactate, NPY, or cortisol. DHEA levels increased from baseline to post-training in week 1, but decreased from baseline to post-training in week. Comparison between baseline timepoints at weeks 1 and 2 revealed that DHEA levels were higher in the beginning of week 2 than at the beginning of week 1. No other comparison between these two time points reached statistical significance for the remaining blood biomarkers. The testosterone/cortisol ratio was shown to be higher at baseline than at post-training There was no effect for Week or a Week × Timepoint interaction		*p* > 0.05 *p* < 0.05, partial eta-squared = 0.55 *p* < 0.05 *p* > 0.05 *p* = 0.05, partial eta squared = 0.36
Vicente-Rodriquez et al., 2020 [54]	Underwater evacuation training	2 h, 4 × 30 min events	1. RPE 2. Subjective Stress Perception 3. Short-term memory 4. BOS (%) 5. Mean HR (bpm) 6. Cortical Arousal (Hz) 7. HRV—RMSSD 8. HRV—HF 9. HRV—LF	All variables were assessed previous to and immediately after evacuation training	1. 6.36 ± 1.10 2. 22.17 ± 23.04 3. 1.00 ± 0.0 4. 96.31 ± 1.80 5. 88.45 ± 14.69 6. 39.35 ± 3.59 7. 33.11 ± 18.73 8. 20.85 ± 11.58 9. 79.08 ± 11.63	1. 12.47 ± 2.99 2. 56.25 ± 26.36 3. 1.00 ± 0.0 4. 96.17 ± 2.49 5. 97.68 ± 13.36 6. 37.94 ± 3.70 7. 35.71 ± 18.93 8. 23.74 ± 10.02 9. 76.15 ± 10.09	1. *p* = 0.000 2. *p* = 0.000 3. NR 4. *p* = 0.801 5. *p* = 0.000 6. *p* = 0.100 7. *p* = 0.000 8. *p*−0.024 9. *p* = 0.023
Wilkinson et al., 2019 [55]	SCBA Confidence course and live-fire training vs. circuit training	Circuit training (CT), SCBA confidence course (SCBA), and live-fire training drills (LFT) each lasted ~33–38 min	1. HR peak (bpm) 2. HR mean (bpm) 3. Estimated core temperature (°C)	Variables were collected continuously throughout events	1. CT: 183 ± 9, SCBA: 193 ± 7 *, LFT: 195 ± 10 *^ 2. CT: 155 ± 10, SCBA: 163 ± 12 *, LFT: 165 ± 10 * 3. CT: 38.6 ± 0.4, SCBA: 39.4 ± 0.3 *, LFT: 39.3 ± 0.4 *	No post-training values reported—only values during training	* *p* <0.001 vs. CT *^ *p* < 0.01 vs. SCBA
Young et al., 2014 [56]	Self-contained breathing apparatus tasks, to include free search (FS), guideline search (GS), and live firefighting (LF) tasks under room temp and extreme heat	Mean time to complete tasks: 17 min for free-search, 14 min for guidelines, and 19 min for live firefighting	1. HR (bpm) 2. RPE 3. NASA Task Load Index (mental demand) 4. Bond-Lader Mood Scale (calmness)	1 & 4. Recorded after safety brief before exercise All variables recorded aftet completion of tasks	1. FS: 91 ± 21, GS: 84 ± 11, LF: 70 ± 8 2. NR 3. NR 4. FS: 69 ± 19, GS: 67 ± 16, LF: 71 ± 16	1. FS: 104 ± 18, GS: 104 ± 19, LF: 98 ± 12 2. FS: 13 ± 1.7, GS: 13 ± 1.9, LF: 15 ± 2.1 3. FS: 74 ± 22, GS: 72 ± 11, LF: 75 ± 19 4. FS: 57 ± 13, GS: 68 ± 17, LF: 55 ± 19	1. FS: *p* = 0.005, GS: *p* = 0.001, LF: *p* < 0.0001 2. NR 3. NR 4. *p* < 0.01 for all
Zare et al., 2018 [57]	Live-fire activities (LFA), typical firefighting activites (TFA), and rescue operations at height (ROH)	20–25 min for each activity	1. HR (bpm) 2. Mean temporal temperature (°C) 3. Paced auditory test	1. Measured at baselline and continiously throughout with HR monitor 2. Measured prior to and at the end of each scenario 3. Adminstered before and after each scenario	1. LFA: 69.8 ± 6.2, TFA: 69.8 ± 5.7, ROH: 70.0 ± 5.9 2. LFA: 37.1 ± 0.12, TFA: 37.1 ± 0.1, ROH: 37.1 ± 0.09 3. LFA: 50.3 ± 1.1, TFA: 50.2 ± 1.1, ROH: 50.4 ± 1.5	1. LFA: 149.3 ± 3.7, TFA: 152.2 ± 3.9, ROH: 159.2 ± 4.1 2. LFA: 38.0 ± 0.06, TFA: 38.1 ± 0.09, ROH: 38.2 ± 0.08 3. LFA: 48.61.2, TFA: 47.7 ± 1.2, ROH: 47.0 ± 1.1	1. *p* < 0.05 for all conditions 2. *p* < 0.05 for all conditions 3. *p* < 0.05 for all conditions

## Data Availability

All data is available.

## Supplementary Materials

The following supporting information can be downloaded at: https://www.mdpi.com/article/10.3390/healthcare11182515/s1, Table S1: Articles excluded from the review with reasons. Table S2: General trends of measures and volume of evidence. References [99,100,101,102,103,104,105,106,107,108,109,110,111,112,113,114] are cited in the supplementary materials.
