# Supplementary material for: An Examination of Subjective and Objective Measures of Stress in Tactical Populations: A Scoping Review
Source: Healthcare (Basel). 2023 Sep 11;11(18):2515. doi: 10.3390/healthcare11182515 (PMC10530665; doi:10.3390/healthcare11182515)
Supplement: Supplementary file 1 [file healthcare-11-02515-s001.zip › Supplemental Table S1 Excluded Articles.pdf]

Supplemental Table S1: Articles excluded from the review with reasons

Reason for exclusion	References
Stress in cognitive tasks	[2, 6, 15, 17]
Conducted during extreme temperature (hot or cold)	[9-13]
Fitness measures used as predictors of stress	[1,8]
Anthropometric measures used as predictors of stress	[14]
Examined long-term deployment	[7]
Measures collected during graded exercise test	[16]
Outcome measures used to quantify stress	[4]
Stress as measure of infection	[5]
Measures collected to predict selection	[3]

References:

1. Abel, M. G., Mortara, A. J., & Pettitt, R. W. (2011). Evaluation of circuit-training intensity for firefighters. *Journal of Strength and Conditioning Research*, *25*(10), 2895–2901.
2. Bhattacharyya, D., Pal, M., Chatterjee, T., & Majumdar, D. (2017). Effect of load carriage and natural terrain conditions on cognitive performance in Desert Environments. *Physiology & Behavior*, *179*, 253–261.
3. Farina, E. K., Thompson, L. A., Knapik, J. J., Pasiakos, S. M., McClung, J. P., & Lieberman, H. R. (2019). Physical performance, demographic, psychological, and physiological predictors of success in the U.S. Army Special Forces Assessment and Selection Course. *Physiology & Behavior, 210*, 112647.
4. Gomes, P., Kaiseler, M., Lopes, B., Faria, S., Queiros, C., & Coimbra, M. (2013). Are standard heart rate variability measures associated with the self-perception of stress of firefighters in action? *2013 35th Annual International Conference of the IEEE Engineering in Medicine and Biology Society (EMBC)*.
5. Gomez-Merino, D., Drogou, C., Chennaoui, M., Tiollier, E., Mathieu, J., & Guezennec, C. Y. (2005). Effects of combined stress during intense training on cellular immunity, hormones and respiratory infections. *Neuroimmunomodulation*, *12*(3), 164–172.
6. Hansen, A. L., Johnsen, B. H., & Thayer, J. F. (2009). Relationship between heart rate variability and cognitive function during threat of shock. *Anxiety, Stress & Coping*, *22*(1), 77–89.
7. Hellewell, S. C., & Cernak, I. (2018). Measuring resilience to operational stress in Canadian Armed Forces personnel. *Journal of Traumatic Stress, 31*(1), 89-101.
8. Hormeño-Holgado, A. J., Nikolaidis, P. T., & Clemente-Suárez, V. J. (2019). Psychophysiological patterns related to success in a special operation selection course. *Frontiers in Physiology*, *10*.
9. Larsen, B., Snow, R., & Aisbett, B. (2015). Effect of heat on firefighters' work performance and physiology. *Journal of Thermal Biology*, *53*, 1–8.
10. Larsen, B., Snow, R., Vincent, G., Tran, J., Wolkow, A., & Aisbett, B. (2015). Multiple days of heat exposure on firefighters' work performance and physiology. *PLOS ONE*, *10*(9).
11. Larsen, B., Snow, R., Williams-Bell, M., & Aisbett, B. (2015). Simulated firefighting task performance and physiology under very hot conditions. *Frontiers in Physiology*, *6*.
12. Petruzzello, S. J., Gapin, J. I., Snook, E., & Smith, D. L. (2009). Perceptual and physiological heat strain: Examination in firefighters in laboratory- and field-based studies. *Ergonomics*, *52*(6), 747–754.

13. Prell, R., Opatz, O., Merati, G., Gesche, B., Gunga, H.-C., & Maggioni, M. A. (2020). Heart rate variability, risk-taking behavior and resilience in firefighters during a simulated extinguish-fire task. *Frontiers in Physiology*, *11*.
14. Sal, M. D., Barbieri, E., Garbati, P., Sisti, D., Rocchi, M. B., & Stocchi, V. (2009). Physiologic responses of firefighter recruits during a supervised live-fire work performance test. *Journal of Strength and Conditioning Research*, *23*(8), 2396–2404.
15. Taverniers, J., Smeets, T., Van Ruysseveldt, J., Syroit, J., & von Grumbkow, J. (2011). The risk of being shot at: Stress, cortisol secretion, and their impact on memory and perceived learning during reality-based practice for armed officers. *International Journal of Stress Management*, *18*(2), 113–132.
16. Von Heimburg, E., Sandsund, M., Rangul, T. P., & Reinertsen, R. E. (2017). Physiological and perceptual strain of firefighters during graded exercise to exhaustion at 40 and 10 °C. *International Journal of Occupational Safety and Ergonomics*, *25*(3), 412–422.
17. Watt, P. W., Willmott, A. G. B., Maxwell, N. S., Smeeton, N. J., Watt, E., & Richardson, A. J. (2016). Physiological and psychological responses in fire instructors to heat exposures. *Journal of Thermal Biology*, *58*, 106–114.
