# Supplementary material for: An Examination of Subjective and Objective Measures of Stress in Tactical Populations: A Scoping Review
Source: Healthcare (Basel). 2023 Sep 11;11(18):2515. doi: 10.3390/healthcare11182515 (PMC10530665; doi:10.3390/healthcare11182515)
Supplement: Supplementary file 1 [file healthcare-11-02515-s001.zip › Supplemental Table S2.pdf]

Supplemental Table S2: General trends of measures and volume of evidence

		**Overall trend**	**n of studies**	**References**
Physiological	HR	⇧	24	1, 5-7, 9-11, 14, 15, 17-19, 25-29, 31, 33-35, 38-42
	HRV - RMSSD	⇧	9	7, 14, 18, 19, 27, 29, 33, 35, 39
	HRV - HF	⇧⇩	7	19, 27, 29, 31, 33, 35, 39
	HRV - LF	⇧⇩	7	19, 27, 29, 31, 33, 35, 39
	Body Temp	⇧	14	2, 7, 9-11, 15, 25, 27, 28, 31, 33, 34, 40, 42
	SpO_2	⇧⇩	9	2, 5, 9, 27-29, 33, 34, 39
Saliva	Cortisol	⇧	9	1, 3, 5, 12, 13, 17, 24, 26, 32
	Alpha Amylase	⇧	1	24
	CRP	⇔	2	12, 13
	NPY	⇧	1	17
	DHEA	⇧	2	17, 32
	Testosterone	⇧	1	17
Blood	$[La^-]_b$	⇧	11	2, 5, 6, 19, 27-29, 33-35, 38
	CK	⇧	3	4, 5, 23
	Cortisol	⇧	8	16, 17, 21, 22, 30, 36-38
	Testosterone	⇩	6	21, 22, 30, 36-38
	IGF-1	⇩	4	21-23, 37
	SHBG	⇧	4	8, 22, 36, 37
	DHEA	⇧	3	16, 17, 38
	IL-6	⇧	2	4, 38
	CRP	⇧	1	8
	NPY	⇔	1	30, 38
	sTfR	⇧	1	17
	Hepcidin	⇧	1	20
	TNF-a	⇩	1	23
	BDNF	⇔	1	16
	Epinephrine	⇧	2	17, 30
	Norepinephrine	⇧	2	17, 30
	Dopamine	⇧	1	30
	TSH	⇔	1	8
	Leptin	⇩	1	23
	T3	⇩	1	8
	T4	⇩	1	8
	Glucose	⇧	4	5, 7, 28, 29
Psychological	RPE	⇧	14	2, 6, 7, 9, 15, 18, 19, 25, 27, 28, 33, 35, 39, 41

STAI	⇧	10	2, 9, 24-29, 33, 35
CSAI-2R	⇧⇩	7	2, 5, 27, 28, 29, 33, 35
POMS	⇧⇩	4	4, 17, 24, 38
SSP	⇧	3	2, 9, 39
DASS	⇧	1	4
ERIQ	⇔	2	12, 13
Perceptions of thermal and respiratory distress	⇧	1	25
IES-R	⇧	1	32

1. Backe, E.M., et al., *Assessment of salivary cortisol as stress marker in ambulance service personnel: comparison between shifts working on mobile intensive care unit and patient transport ambulance.* Int Arch Occup Environ Health, 2009. **82**(9): p. 1057-64.
2. Bustamante-Sanchez, A. and V.J. Clemente-Suarez, *Psychophysiological response in night and instrument helicopter flights.* Ergonomics, 2020. **63**(4): p. 399-406.
3. Charles, L.E., et al., *Shiftwork and Diurnal Salivary Cortisol Patterns Among Police Officers.* J Occup Environ Med, 2016. **58**(6): p. 542-9.
4. Chester, A.L., et al., *Physiological, biochemical, and psychological responses to environmental survival training in the Royal Australian Air Force.* Mil Med, 2013. **178**(7): p. e829-35.
5. Clemente-Suarez, V.J., et al., *Experience modulates the psychophysiological response of airborne warfighters during a tactical combat parachute jump.* Int J Psychophysiol, 2016. **110**: p. 212-216.
6. Clemente-Suarez, V.J., P.R. Palomera, and J.J. Robles-Perez, *Psychophysiological response to acute-high-stress combat situations in professional soldiers.* Stress Health, 2018. **34**(2): p. 247-252
7. Gomez-Oliva, E., et al., *Psychophysiological response to the use of nuclear, biological and chemical equipment with military tasks.* Physiol Behav, 2019. **204**: p. 186-190.
8. Hamarsland, H., et al., *Depressed Physical Performance Outlasts Hormonal Disturbances after Military Training.* Med Sci Sports Exerc, 2018. **50**(10): p. 2076-2084.
9. Hormeno-Holgado, A.J. and V.J. Clemente-Suarez, *Psychophysiological Monitorization in a Special Operation Selection Course.* J Med Syst, 2019. **43**(3): p. 47.
10. Horn, G.P., et al., *Firefighter and fire instructor's physiological responses and safety in various training fire environments.* Safety Science, 2019. **116**: p. 287-294.
11. Hunt, A.P., I.B. Stewart, and D.C. Billing, *Indices of physiological strain for firefighters of the Australian Defence Forces.* J Occup Environ Hyg, 2019. **16**(11): p. 727-734.
12. Iizuka, N., S. Awano, and T. Ansai, *Salivary alpha-amylase activity and stress in Japan air self-defense force cargo pilots involved in Iraq reconstruction.* Am J Hum Biol, 2012. **24**(4): p. 468-72.
13. Izawa, S., A. Tsutsumi, and N. Ogawa, *Effort-reward imbalance, cortisol secretion, and inflammatory activity in police officers with 24-h work shifts.* Int Arch Occup Environ Health, 2016. **89**(7): p. 1147-54.
14. Kaikkonen, P., H. Lindholm, and S. Lusa, *Physiological Load and Psychological Stress During a 24-hour Work Shift Among Finnish Firefighters.* J Occup Environ Med, 2017. **59**(1): p. 41-46.
15. Kesler, R.M., et al., *Physiological response to firefighting activities of various work cycles using extended duration and prototype SCBA.* Ergonomics, 2018. **61**(3): p. 390-403.
16. Ledford, A.K., et al., *Psychological and Physiological Predictors of Resilience in Navy SEAL Training.* Behav Med, 2020. **46**(3-4): p. 290-301.

17. Lieberman, H.R., et al., *Cognitive function, stress hormones, heart rate and nutritional status during simulated captivity in military survival training.* Physiol Behav, 2016. **165**: p. 86-97.
18. Marcel-Millet, P., et al., *Physiological responses and parasympathetic reactivation in rescue interventions: The effect of the breathing apparatus.* Scand J Med Sci Sports, 2018. **28**(12): p. 2710-2722.
19. Marins, E.F., et al., *Effects of personal protective equipment on metabolism and performance during an occupational physical ability test for federal highway police officers.* JSCR, 2020. **34**: p. 1093-1102.
20. McClung, J.P., et al., *Effects of a 7-day military training exercise on inflammatory biomarkers, serum hepcidin, and iron status.* Nutrition Journal 2013. **12**.
21. Nindl, B.C., et al., *Physiological consequences of U.S. Army Ranger training.* Med Sci Sports Exerc, 2007. **39**(8): p. 1380-7.
22. Ojanen, T., et al., *Effect of Prolonged Military Field Training on Neuromuscular and Hormonal Responses and Shooting Performance in Warfighters.* Mil Med, 2018. **183**(11-12): p. e705-e712.
23. Ojanen, T., P. Jalanko, and H. Kyrolainen, *Physical fitness, hormonal, and immunological responses during prolonged military field training.* Physiol Rep, 2018. **6**(17): p. e13850.
24. Perroni, F., et al., *Effects of simulated firefighting on the responses of salivary cortisol, alpha-amylase and psychological variables.* Ergonomics, 2009. **52**(4): p. 484-91.
25. Petruzzello, S.J., et al., *Physiological, Perceptual and Psychological Responses of Career versus Volunteer Firefighters to Live-fire Training Drills.* Stress Health, 2016. **32**(4): p. 328-336.
26. Regehr, C., et al., *Acute stress and performance in police recruits.* Stress and Health, 2008. **24**(4): p. 295-303.
27. Sanchez-Molina, J., J.J. Robles-Perez, and V.J. Clemente-Suarez, *Assessment of Psychophysiological Response and Specific Fine Motor Skills in Combat Units.* J Med Syst, 2018. **42**(4): p. 67.
28. Sanchez-Molina, J., J.J. Robles-Perez, and V.J. Clemente-Suarez, *Psychophysiological and Specific Fine Motor Skill Modifications in a Checkpoint Action.* J Med Syst, 2019. **43**(4): p. 90.
29. Sanchez-Molina, J., J.J. Robles-Perez, and V.J. Clemente-Suarez, *Effect of Parachute Jump in the Psychophysiological Response of Soldiers in Urban Combat.* J Med Syst, 2017. **41**(6): p. 99.
30. Szivak, T.K., et al., *Adrenal Stress and Physical Performance During Military Survival Training.* Aerosp Med Hum Perform, 2018. **89**(2): p. 99-107.
31. Takeyama, H., et al., *Effects of shift schedules on fatigue and physiological functions among firefighters during night duty.* Ergonomics, 2005. **48**(1): p. 1-11.
32. Taylor, M.K., et al., *Stressful military training: endocrine reactivity, performance, and psychological impact.* Aviat Space Environ Med, 2007. **78**(12): p. 1143-9.
33. Tornero-Aguilera, J.F. and V.J. Clemente-Suarez, *Effect of experience, equipment and fire actions in psychophysiological response and memory of soldiers in actual underground operations.* Int J Psychophysiol, 2018. **128**: p. 40-46.
34. Tornero-Aguilera, J.F., J.J. Robles-Perez, and V.J. Clemente-Suarez, *Effect of Combat Stress in the Psychophysiological Response of Elite and Non-Elite Soldiers.* J Med Syst, 2017. **41**(6): p. 100.
35. Tornero-Aguilera, J.F., J.J. Robles-Perez, and V.J. Clemente-Suarez, *Use of Psychophysiological Portable Devices to Analyse Stress Response in Different Experienced Soldiers.* J Med Syst, 2018. **42**(4): p. 75
36. Tyyska, J., et al., *Association with physical fitness, serum hormones and sleep during a 15-day military field training.* J Sci Med Sport, 2010. **13**(3): p. 356-9.
37. Vaara, J.P., et al., *Physical Fitness and Hormonal Profile During an 11-Week Paratroop Training Period.* JSCR, 2015. **29**.
38. Vartanian, O., et al., *Changes in mood, fatigue, sleep, cognitive performance and stress hormones among instructors conducting stressful military captivity survival training.* Physiol Behav, 2018. **194**: p. 137-143.
39. Vicente-Rodriguez, M., J.P. Fuentes-Garcia, and V.J. Clemente-Suarez, *Psychophysiological Stress Response in an Underwater Evacuation Training.* Int J Environ Res Public Health, 2020. **17**(7).
40. Wilkinson, A.F., et al., *Physiologic strain of SCBA confidence course training compared to circuit training and live-fire training.* Appl Ergon, 2020. **82**: p. 102966.
41. Young, P.M., et al., *Psychophysiological responses in experienced firefighters undertaking repeated self-contained breathing apparatus tasks.* Ergonomics, 2014. **57**(12): p. 1898-906.
42. Zare, S., et al., *Comparison of the effect of typical firefighting activities, live fire drills and rescue operations at height on firefighters' physiological responses and cognitive function.* Ergonomics, 2018. **61**(10): p. 1334-1344.
